# Cocoa Bean Shell—A By-Product with Nutritional Properties and Biofunctional Potential

**DOI:** 10.3390/nu12041123

**Published:** 2020-04-17

**Authors:** Olga Rojo-Poveda, Letricia Barbosa-Pereira, Giuseppe Zeppa, Caroline Stévigny

**Affiliations:** 1 RD3 Department-Unit of Pharmacognosy, Bioanalysis and Drug Discovery, Faculty of Pharmacy, Université libre de Bruxelles, 1050 Brussels, Belgium; 2 Department of Agriculture, Forestry and Food Sciences (DISAFA), University of Turin, 10095 Grugliasco, Italy; 3 Department of Analytical Chemistry, Nutrition and Food Science, Faculty of Pharmacy, University of Santiago de Compostela, 15782 Santiago de Compostela, Spain; letricia.barbosa.pereira@usc.es

**Keywords:** cocoa by-product, biofunctional, bioactivity, polyphenols, flavonoids, methylxanthines, theobromine, antibacterial, anticarcinogenic, antidiabetic

## Abstract

Cocoa bean shells (CBS) are one of the main by-products from the transformation of cocoa beans, representing 10%‒17% of the total cocoa bean weight. Hence, their disposal could lead to environmental and economic issues. As CBS could be a source of nutrients and interesting compounds, such as fiber (around 50% *w*/*w*), cocoa volatile compounds, proteins, minerals, vitamins, and a large spectrum of polyphenols, CBS may be a valuable ingredient/additive for innovative and functional foods. In fact, the valorization of food by-products within the frame of a circular economy is becoming crucial due to economic and environmental reasons. The aim of this review is to look over the chemical and nutritional composition of CBS and to revise the several uses that have been proposed in order to valorize this by-product for food, livestock feed, or industrial usages, but also for different medical applications. A special focus will be directed to studies that have reported the biofunctional potential of CBS for human health, such as antibacterial, antiviral, anticarcinogenic, antidiabetic, or neuroprotective activities, benefits for the cardiovascular system, or an anti-inflammatory capacity.

## 1. Introduction

**Figure 1:**
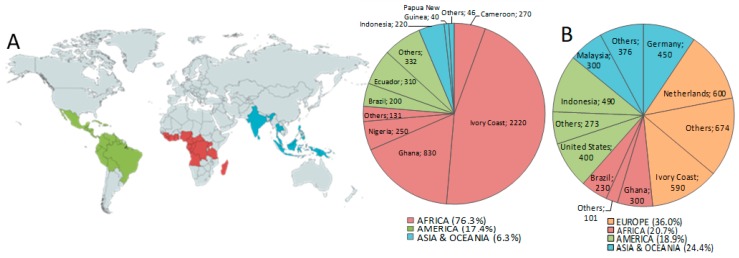
Forecast of global cocoa bean production (**A**) and global cocoa grinding (**B**) during the season of 2018/2019. Adapted from [2].

One part of the fruit from the plant *Theobroma cacao* L. is the well-known cocoa bean, which is the main raw material for chocolate manufacturing. Cocoa bean production takes place mainly in tropical areas, and it reaches more than 4.7 million tons per year worldwide, from which 76.3%, 17.4%, and 6.3%, were estimated to be produced in Africa, America, and Asia and Oceania, respectively, during the harvest season of 2018/2019 (Figure 1A). Cocoa bean exportation constitutes about 71% of the total produced volume [1], and, although Europe is not a producing continent, its processing of cocoa beans reaches 1.7 million tons, leading the statistics over other continents [2] (Figure 1B). After being harvested, cocoa beans are first separated from their pods, then they are subjected to fermentation, followed by a drying phase. At this point, cocoa beans are transferred to the chocolate production industries, where they are roasted and winnowed in order to separate them from their shells, since no more than a 5% of shell is allowed on cocoa products according to the Codex Alimentarius [3,4,5].

**Figure 2:**
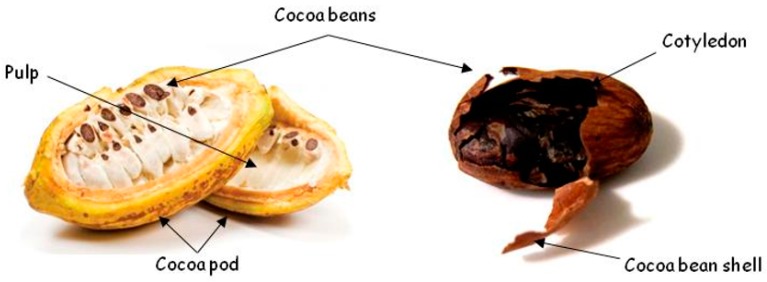
Cocoa beans and their processing by-products. Adapted from [10,11].

Cocoa production generates substantial quantities of waste. Indeed, only 10% of the total cocoa fruit weight is used for its commercialization, while the remaining 90% is discarded as waste or by-products [6,7]. One of these by-products is the external tegument that cover the cocoa beans, also known as cocoa bean shells (CBS; Figure 2), which are generated during the cocoa bean roasting process, as already mentioned. CBS constitute about 10%–17% of the total cocoa bean weight [8] and some studies have revealed that these percentages are likely to vary depending on the fermentation type of cocoa beans [9].

Taking into account the weight percentage of CBS and the aforementioned cocoa production data, this would mean that more than 700 thousand tons of CBS waste is produced worldwide, from which more than 250 thousand tons is only produced in Europe. To give an idea of this, the production of one kg of chocolate would produce an output of 98 g of CBS [12]. The increasing demand for cocoa beans has led to an accumulation of this by-product, representing a serious disposal problem that could be aggravated by legal restrictions [7]. Indeed, the disposal of CBS could carry important economic and environmental issues [13], as they contain polyphenols with potential phytotoxic activity [14] and considerable amounts of theobromine, which has been reported to be toxic for some non-human mammals [15]. Moreover, its toxicity in aquatic animals has also been reported [16].

Despite being considered a by-product, the nutritional composition of CBS does not differ hugely from that of cocoa beans, except for fats, which are much more present in cocoa beans, while fibers predominate the shells [17]. Besides, CBS also contains considerable quantities of interesting bioactive compounds, such as polyphenols, which are known to be responsible for the different nutrition-related health benefits provided by cocoa [18].

Recently, the bioconversion of food processing residues into valuable products has begun to receive increasing attention, and as a result, industrial countries are preparing strategic policies to develop a bio-based circular economy [19,20]. Due to all the aforementioned reasons, valorization strategies for CBS have appeared in different fields, and several studies have been carried out in order to find new applications for this by-product. Among these applications, new uses in the food industry field, feedstuff for livestock, or utilization by industry as a biofuel, absorbent or composite, among others, could be considered as the most commons applications. Detailed reviews regarding these applications have already been done by Okiyama et al. [21] and Panak Balentić et al. [22]. However, in the last few years, other types of applications focusing on the biofunctionality and bioactivity of this cocoa by-product have appeared. Therefore, the aim of this literature review is to look over the current knowledge and latest advances of CBS applications for human health from a nutritional and biofunctional point of view, while other applications will only be briefly reviewed.

## 2. Methods and Literature Search

Peer-reviewed literature and books published between 2000 and 2020 were examined and employed for the elaboration of this review; however some works prior to that time frame were eventually included for being considered relevant in the field or for being the latest known studies for a particular aspect of the research field. Literature was examined, using the following databases and search engines: Scopus, the Web of Science, SciFinder, ResearchGate, Google Scholar, PubMed, and SciELO. Scientific papers and other sources were also selected and found manually by analyzing the bibliographies of all the collected articles. Then, the literature was obtained mainly by downloading it through Google Scholar as the principal source, among others. Different search terms were used individually and in combination, such as “polyphenols”, “theobromine”, “biofunction”, “cocoa”, “theobroma”, “by-product”, or “cocoa bean shell”. Also, different synonyms for the cocoa bean shell term were employed in order to complete the research, such as husk, hull, testa, or tegument.

## 3. Nutritional and Chemical Composition

### 3.1. Proximate composition—Moisture, Ashes, Proteins, Fats, and Carbohydrates

The proximate composition of CBS has been reported by several authors and this is summarized in Table 1. CBS proximate composition comprises proteins, fats, sugars, moisture, and ashes [23], and has been described to be similar to that of cocoa beans. However, CBS present a much lower percentage of fats compared to cocoa beans, which is substituted by a much higher amount of fibers [17]. CBS also have a higher content of proteins, fats, and carbohydrates compared to other cocoa by-products, such as cocoa pods [24].

However, the proximate composition of CBS can significantly vary, since, as a vegetable product, its composition is subjected to several variable factors, such as the climatic conditions of the farming area, the cocoa variety, processing conditions (fermentation, drying, roasting temperature), etc. [25]. 

The values found for the moisture of CBS range from 3.60% to 13.13%, which highly depends on whether the CBS are roasted or not [26]. Bonvehí et al. [27] obtained values between 3.6% and 7.8% for moisture and affirmed that this is an acceptable range for stable CBS storage. Nonetheless, CBS have been reported to be considerably hygroscopic, and, therefore, molds could appear if stored at higher moisture levels [13,28].

Ash content was established to be between 5.96 and 11.42 g/100 g of CBS according to the literature, once again being influenced by the roasting process, which increases this value by about 15% according to Agus et al. [26]. Osundahunsi et al. [28] reported that the main components found in CBS ash are sodium and potassium (7.2 g and 3.1 g per 100 g of CBS ash, respectively). Of the total calculated ash, Gónzalez et al. [1] reported that 30.4% of it would be water-soluble ash while about 38.4% would be acid-insoluble ash (mainly silica derivates such as sand and siliceous earth) [29].

Concerning the protein content, the reviewed literature establishes that proteins constitute between 10.30% and 27.40% of the CBS dried weight. This is a remarkable quantity and, therefore, some researchers have considered this by-product as a source of extractable protein [36,42]. In addition, one study has found that fermenting the shells with *Pleurotus ostreatus* spawn could produce an increase of up to 25.2% in the protein content [69]. However, it has been shown that the roasting process normally has an unfavorable effect on this quantity, as Agus et al. [26] reported a decrease from 27.43% to 25.07% of crude protein for CBS after roasting. Pérez et al. [24] found that CBS proteins have 78.04% digestibility, which is not far from the 68% reported by Bonvehí et al. [27]. CBS contain all the essential amino acids, representing 44.7% of the total amino acids [27,52]. However, a small percentage of the total amino acids are D-amino acids, and their relative quantity with respect to L-amino acids increases during the roasting phase. D-amino acids provide low nutritional value, as they are not digested like their chiral counterparts. Nevertheless, they still contribute to flavor formation during fermentation and roasting [70].

The fat content accounts for 1.50%‒8.49% of dried CBS and is therefore considered a minor component of the by-product when compared to the approximate 50% fat content in cocoa beans [27]. However, the fat in CBS has also received interest from researchers that have optimized methods for its extraction [71]. As reported for the protein content, the roasting process could also entail a decrease of about 36% of the fat in CBS [26]. As CBS fat is highly acidic and richer in the unsaponifiable matter than cocoa bean fat, it is not often considered as cocoa butter [45,60]. Indeed, some CBS fat compounds differ considerably from those of cocoa butter, and, in some cases, these differences have been used in order to estimate the shell content of cocoa powder [72] or cocoa butter [73]. Nevertheless, oleic, palmitic, capric, and stearic acids are the main fatty acids in both CBS and cocoa fats when considering the saponifiable fraction [45,57,74]. Regarding this fraction, Lessa et al. [75] found that it is comprised of 34.7% unsaturated fatty acids and 64% saturated fatty acids for non-fermented CBS, and that these percentages vary to 51.2% and 48%, respectively, after fermentation. These values are in accordance with the 0.66‒0.74 unsaturated/saturated mass ratio reported by Okiyama et al. [36]. Also, phytoprostanes and phytofurans, which are isoprostanoids derived from the peroxidation of α-linoleic acid, have been detected in CBS in quantities of 474.3 and 278.0 ng per gram of dried CBS, respectively [76]. The unsaponifiable fraction of the CBS fat is formed by compounds such as phytosterols, of which stigmasterol would be the predominant one, while cholesterol concentrations are almost insignificant when compared to those of cocoa beans [26].

According to the literature, carbohydrates constitute 7.85%‒70.25% of the CBS dry weight. These values differ considerably depending on whether fiber content is taken into consideration or not, and also because they are often calculated by subtraction, which entails added variability [35]. Concerning digestible carbohydrates, CBS contain none or small quantities of starch, mostly available starch [24], and a very small quantity of soluble sugars, considered negligible in some studies [27,40]. Regarding the non-digestible fraction, this is formed by pectic polysaccharides (45%), hemicelluloses (20%), and cellulose (35%), and constitutes the dietary fiber [77], which will be explained in detail in Section 3.2. Glucose is the main monosaccharide in CBS and accounts for almost half of the carbohydrate fraction, followed by galactose, mannose, rhamnose, arabinose, and xylose in a decreasing order [32,52,74,77].

### 3.2. Dietary Fiber

The dietary fiber of CBS is composed of structural carbohydrates, also known as non-starch polysaccharides. It is constituted by residues of plant cell walls and is not digestible by human enzymes; therefore, it provides no energy value [32,33]. Consumption of dietary fiber is important due to its contribution to proper intestinal transit. Some authors have also reported that the dietary fiber contained in CBS possesses several other biofunctions, such as reducing cardiovascular risks by reducing cholesterol and triacylglycerol levels or reducing diabetes effects by retarding glucose absorption (as reviewed in Section 5 of this paper) [78,79]. On the other hand, CBS dietary fiber also adsorbs important concentrations of polyphenols, which could give it antioxidant properties and contribute to decreasing oxidative stress and inflammation processes in the intestine [7,80].

The total dietary fiber (TDF), soluble dietary fiber (SDF), and insoluble dietary fiber (IDF) values of CBS reported in the literature are shown in Table 1. Noteworthy differences for these values are due to the way CBS fiber has been determined in the different studies. Gravimetric methods that are usually used for fiber analysis comprise both the non-starch polysaccharide fraction and the fraction known as the ‘Klason lignin’ fraction, which is in some cases formed not only by lignin, but also by Maillard products and complexes formed by tannin and protein interactions [77]. Redgwell et al. found that gravimetrically determined fiber on CBS (including the Klason fraction) accounts for 63.6% of the CBS dried weight, while fiber determined as total polysaccharides would be just 38.2% [77]. Similarly, Lecumberri et al. found values of 60.5% and 28.1%, respectively [40]. Excluding the Klason fraction, CBS fiber would be composed of about 45% pectic compounds, 35% celluloses, and 20% hemicelluloses. As mentioned in Section 3.1, glucose would be the main monosaccharide composing CBS fiber. Calculations of the IDF and SDF fractions on CBS vary between authors, however, IDF is always the most abundant, with the IDF/SDF ratio ranging between 2.2 and 4 [35,38,77]. Compared to other cocoa by-products, such as the cocoa pod, CBS possess equivalent quantities of total dietary fiber, but with a higher percentage of SDF, which would be the one providing more interesting biofunctional properties, as described by Matínez et al. [35].

Particularly, the pectin fraction of CBS fiber formed mainly by galacturonic acids has attracted the attention of many researchers, mainly because of its interesting gelling properties, which are very useful in fields such as the food, pharmaceutical, or cosmetic industries [30,49]. Pectins are present in both SDF and IDF in the form of high methoxyl pectins for the former and low methoxyl pectins for the latter [17]. CBS pectin is sometimes considered a “low quality pectin” when compared to other commercial pectins [51,81] and is present in lower concentrations than that of citrus or apples (about 9% against 15% and 30% dry weight, respectively) [50]. However, optimizations for the pectin extraction process from CBS have been proposed [49,82].

### 3.3. Phenolic Compounds

Together with fiber, polyphenols are the most interesting and studied compounds in CBS and are the main compounds responsible for the biofunctional properties attributed to this cocoa by-product. These compounds are present in all vegetable origin foods and they are well known for producing several biological activities. A special group of polyphenols are flavonoids, among which, flavanols are the main group in cocoa [83]. They are not essential for short-term well-being, but there is growing evidence suggesting that a modest long-term intake of polyphenols could give several health benefits, as they possess antioxidant properties, act as free-radical scavengers, and reduce oxidative stress. They can take part in anti-inflammatory processes, exert antidiabetic properties, or reduce the risk of several diseases such as cancer, chronic diseases, cardiovascular disease, or even neurodegenerative disorders [80,84,85,86,87,88]. In addition, the intake of dietary flavanols has been reported to improve cognitive function and task performance [89].

The total phenolic content (TPC; expressed as mg of gallic acid equivalents/g of dried CBS), total flavonoid content (expressed as mg of catechin equivalents/g of dried CBS), and total tannin content (expressed as mg of catechin equivalents/g of dried CBS) reported by several researchers are gathered in Table 1 and range between 6.04‒94.95, 1.65‒40.72, and 1.70‒25.30, respectively. Again, these values show great variability depending on the research work, mainly due to the polyphenolic extraction conditions and the employed solvents, although the total flavonoid content (TFC) and total tannin content (TTC) are in general well correlated with the TPC values [13,59]. Indeed, several authors have taken interest in this fact and have studied several possibilities in order to optimize different types of CBS polyphenol extraction, using techniques such as supercritical CO_2_, water extraction [60,61,63,90,91], pulsed electric fields [59], high-voltage electric discharges [58,66], pressurized ethanol [36,60,64], or ultrasound techniques [92]. Macroporous resins have been used to increase the total polyphenol content of a CBS extract from 2.23% to 62.87% *w*/*w* CBS [93]. The polyphenolic content has been demonstrated to significantly vary depending on the geographic origin, variety, plant genotype, and even the harvest season [59,62]. Bruna et al. attributed a higher polyphenol content to stress situations of the cocoa tree [94]. Other factors affecting polyphenolic quantities could be the type of fermentation and fermentation time, which has been reported to give optimal TPC values after 24 h and then decrease afterwards [75,95]. Light-exposed and high-temperature processes during cocoa manufacturing, such as sun-drying or the roasting process, could imply polyphenol degradation [83]. In order to minimize this kind of degradation and maintain the polyphenolic integrity and activity of CBS, some studies have proposed strategies such as extract encapsulation [42,65].

When compared to cocoa beans, the TPC values of CBS are similar to those between 5.77 and 49.56 mg gallic acid equivalents/g of cocoa beans, as indicated by Hernández-Hernández et al. [96]. Concerning other types of cocoa by-products, it has been found that the TPC values of cocoa pods are slightly higher than those of CBS, while the TFC values are almost 2-fold higher in CBS than in cocoa pods [97]. The antioxidant activity of CBS also seems to be correlated with the total phenolic content of the by-product and some authors have maintained that this activity is mainly due to the flavonoid content of CBS [98], also being influenced by temperature during cocoa processing [99]. However, the TPC, TFC, TTC, and the antioxidant activity are values obtained by screening spectrophotometric methods with several interferences that could vary the obtained quantities, which could also be a reason for the huge ranges of the values that have been found [100].

More specific analyses, such as high-performance liquid chromatography (HPLC) coupled with ultraviolet (UV) or mass spectrometry detection, aiming to find specific polyphenolic compounds on CBS, have shown that procyanidins and catechins are the main polyphenols present in this by-product. In particular, (−)-epicatechin is the most abundant and commonly reported flavan-3-ol contained in CBS, followed by (+)-catechin and their dimmers, procyanidin B1 (epicatechin-(4β → 8)-catechin), and procyanidin B2 (epicatechin-(4β → 8)-epicatechin), which were found in quantities that ranged from 0.21 to 34.97 mg epicatechin/g of CBS, from 0.18 to 4.50 mg catechin/g of CBS, from 0.55 to 0.83 mg procyanidin B1/g of CBS, and from 0.23 to 1.38 mg procyanidin B2/g of CBS (as shown in Table 1). Other polyphenols, such as protocatechuic acid, quercetin and quercetin derivates, caffeic acid, procyanidin dimers, trimers, and tetramers, among others, have also been found in CBS [13,57,59,80,101,102].

### 3.4. Methylxanthines

The main methylxanthines found in CBS are theobromine (3,7-dimethylxanthine) and caffeine (1,3,7-dimethylxanthine). Both are alkaloids that are characteristic of cocoa, although theophylline has also been detected, mostly at trace level, however [63]. Both theobromine and caffeine are known for acting on the central nervous system and influencing mood positively, being one of the reasons for the high cocoa acceptance between consumers. Both methylxanthines have been related to several beneficial effects on human health, such as acting as diuretic, anticarcinogen, or anti-obese agents, among other effects [103]. Caffeine is commonly added to soft drinks as a flavoring agent, and it is also used in pharmaceutical formulations. However, its high consumption has been related to some disorders, such as kidney dysfunction, tachycardia, excessive gastric acid secretion, or even seizures and delirium [104]. On the other hand, theobromine, which is also a caffeine metabolite, is colorless and odorless, with a slightly bitter taste, characteristic of chocolate. Theobromine has a much weaker action on the central nervous system since it has a 2- to 3-fold lower affinity for adenosine receptors than caffeine. Theobromine also possesses myorelaxant and cardiac stimulation properties and has been used as a coronary artery dilator or bronchodilator for asthma treatment [13,105,106,107].

Methylxanthines are mainly synthesized via the cotyledons of cocoa beans [108] and they have been demonstrated to migrate to the shell during cocoa fermentation [109,110]. Indeed, Hernández-Hernández et al. [96] found that theobromine concentrations in raw cocoa beans and raw CBS were 18.07 mg/g of cotyledon and 3.90 mg/g of CBS, respectively, while the concentrations on their fermented counterparts were 9.79 mg/g of cotyledon and 12.00 mg/g of CBS, respectively. The amounts of theobromine in CBS have been reported to be 5‒7-fold higher than caffeine [13]. Concretely, these values were 0.39–1.83 mg/100 g of dried CBS for theobromine and 0.04–0.42 mg/100 g of dried CBS for caffeine. As well as polyphenols, several techniques for methylxanthine extraction optimization from CBS have been proposed [57,58,63,66,67].

Because of these reasons, and also taking into consideration the moderate concentration of caffeine, methylxanthines contained in CBS may also exert interesting bioactivities on human health and could give added value to CBS as a biofunctional ingredient. Moreover, an interaction between cocoa flavanols and methylxanthines has been reported, where methylxanthines help to increase epicatechin levels in plasma, enhancing the vascular effects of flavanols [111].

### 3.5. Minerals and Vitamins

CBS is expected to be rich in minerals because of its considerable quantity of ashes, which represent an index of mineral content in vegetable samples [112]. The mineral amounts found for CBS are reported in Table 1. Potassium, magnesium, calcium, and phosphorous are the most abundant minerals contained in the by-product, followed by smaller quantities of sodium and iron, among others. These elements tend to accumulate in the outer parts of the cocoa bean, and hence they are found in CBS in much higher quantities than in cocoa nibs. Bentil et al. [69] showed that the solid-state fermentation of CBS with *P. ostreatus* spawn and *Aspergillus niger* significantly increased the concentrations of calcium, phosphorus, and potassium. Nevertheless, mineral content in CBS could present great variability, mostly related to the cocoa’s geographic origin, as mineral absorption by the plant is highly dependent on mineral availability in the ground, and is therefore dependent on the soil type and quality of the area [113,114].

Concerning vitamins, CBS have been reported to be a source of vitamin D [115], although studies reporting its concentrations are very old (dating from 1935) and new ones would be necessary. Knapp et al. [54] found quantities up to 21 IU (international units) per gram of CBS (equivalent to 0.53 μg/g of CBS), which is 20–30 times the potency of dairy butter, but only when these were obtained from fermented and sundried cocoa beans, sustaining that vitamin D is probably formed by the light activation of a precursor present in fermentation molds, namely ergosterol. Kon et al. [116] took advantage of this fact and observed that when feeding cows with CBS, vitamin D levels of their butter fat were higher. They also observed that vitamin D was mainly concentrated in CBS fat, which contains 40% of the total vitamin D activity in CBS. Bonvehí et al. [39] found considerable quantities of vitamins B1 and B2 in CBS (shown in Table 1), close to the 15% of the recommended dietary allowance, while vitamins B6 and D were detected only at trace levels and vitamin C was not found in CBS. Also, α-tocopherol, (β + γ)-tocopherol, and δ-tocopherol, which act as vitamin E, were found at a total quantity of 1.02 mg per g of CBS fat.

## 4. Applications

### 4.1. Food Applications

Among its multiple applications, CBS have been largely proposed as clean label ingredients and/or additives because of the nutraceutical character that their high fiber and polyphenol contents provide. Besides, some studies have shown that CBS possess between 10% and 20% of the total amount of volatile organic compounds found in roasted cocoa beans (Table 1), many of them being key aroma compounds for cocoa and chocolate [31,68]. This makes CBS a very interesting, low-cost ingredient for cocoa substitution or cocoa flavoring. For these reasons, CBS have been mainly employed in baked products such as biscuits and bread, in order to increase their fiber content and give them antioxidant properties [48,117,118]. CBS are normally added directly as ground cocoa flour, or as fiber extracts obtained after enzyme treatment. CBS have also been proposed as a fat replacer, replacing up to 50% and 70% of vegetable oil in functional cakes and chocolate muffins, respectively [119,120], with a generally good consumer acceptance in all cases. Another extended use of CBS in the food field has been to create beverages, such as carbonated soft drinks [121], preparations for home-made functional beverages [13], or a dairy drink made with CBS together with other by-products from coffee and oranges [122]. CBS have also been proposed as an extra ingredient to nutritionally fortify extruded snack products, slightly lowering their physical properties, but still within the consumer acceptance range [123].

However, in order to obtain polyphenol-rich products with potential antioxidant capacities, there is concern over the stability of such compounds with temperature or time, or even during the digestion of CBS foods. That is why CBS encapsulation strategies applied to the food field have also been suggested [124]. Altin et al. [125] proposed CBS encapsulation with chitosan-coated liposomes for drinking yogurt preparation that allowed the stabilization of the phenolic content during storage and increased *in vitro* bioaccessibility in terms of the TPC, TFC, and antioxidant activity, while Papillo et al. [65] used CBS microencapsulation by spray-drying with maltodextrins as stabilizing agents in order to stabilize CBS polyphenols in baked products, obtaining biscuits with an invariable polyphenol content after going through the baking process and with up to 90 days of storage.

As a food additive, several authors have taken advantage of CBS antioxidant properties in order to avoid lipid oxidation. To this end, Ismai and Yee [126] added CBS and roselle seed extracts to beef, avoiding lipid oxidation to a greater extent than with synthetic antioxidants such as butylated hydroxytoluene (BHT) and β-tocopherol. Manzano et al. [55] proposed to improve the stability of soya cooking oil by adding a CBS polyphenolic extract, obtaining oils with lower free fatty acids and peroxide generation indices after repeated uses. A similar application was also given by Hernández-Hernández et al. [127], who added an encapsulated CBS polyphenol extract to olive oil jam in order to prevent it from becoming rancid. CBS have also been proposed for the production of liquid smoke additives, which are extensively used for their antioxidant, antibacterial, anti-fungal, anti-termite, and food preservative properties [128]. A different use was proposed by Osundahunsi et al. [28], who suggested to use CBS ashes as an alkalizing agent for cocoa beans, reintroducing the CBS into the chocolate production process. The antioxidant properties of CBS were also used by incorporating them into bioelastomers in order to create active packaging that could preserve foodstuffs for longer [129].

### 4.2. Utilization as Feedstuffs

The use of CBS as a feedstuff has been largely proposed for a long time, as this is common for food processing by-products. CBS have considerable amounts of proteins, minerals, and vitamins which make it an interesting and inexpensive material for livestock feed. However, CBS also contain great quantities of tannins and theobromine, which could act as anti-nutrients in some animals, blocking some essential nutrients during digestion and reducing their bioavailability [130]. Theobromine can also cause different toxic effects in some animals, such as liver and thyroid malfunction in horses [105] or even death in dogs when ingested in high quantities [131].

Despite the presence of theobromine, studies have revealed positive effects when using diets fortified with the right quantity of CBS for poultry, rabbits, ruminants, or pigs. Adeyemo et al. [130,132] fixed the maximum maize and soybean meal substitution with CBS at 10%, where they observed internal organs reduced in weight in broiler birds, while gut morphology was improved, showing enhanced villous and crypt dimensions. Emiola et al. [133] observed the same mentioned negative changes in laying hens with over 15% CBS substitution. Regarding egg quality, maximums of 10%–25% of maize substitution by CBS in laying hens diet was proposed as the limit, beyond which egg weight and quality would be compromised [134,135]. However, an important remark concerning broiler feed with CBS was made by Day and Dilworth [136], who found that equivalent quantities of pure theobromine were more toxic for broilers than those furnished by CBS meal. For rabbits, a maximum substitution of 10% was proposed, as a decrease in the packed cell volume (nutritional deficiencies) and an increase in white blood cells (nutritional stress) was observed above that value, although weight loss was only observed with above 20% CBS meal replacement [43]. Other studies suggested the inclusion of 200 g of CBS per kg of rabbit body weight as the value for the optimal benefit–cost ratio [137,138]. Concerning cattle, a high fiber content supposes an added value for CBS as a feed material, and diets containing up to 40% CBS have been shown to produce positive effects on daily body weight gain and feed efficiency [139]. Used as a bedding material, it was shown that CBS increased milk yield for cows due to an increase in their laying time, additionally, it also decreased bacterial counts on the teat and cortisol levels in cows (which is a stress marker), as well as ammonia concentrations in the barns [140]. Magistrelli et al. [141] showed that a diet with up to 7.5% CBS would not affect growth for pigs and would enhance their microbiota by increasing the levels of bacteria that produce short-chain fatty acids, such as butyrate, known for its anti-inflammatory effects. In aquaculture, it was found that feeding Nile tilapia with a 23% CBS diet resulted in a 35.6% feeding cost reduction and an enhanced weight gain and feed conversion ratio [142]. Other studies have shown that the addition of more than 2315 mg of CBS ethanolic extract per liter of water could cause acute toxicity in Mango tilapia [16].

It has been proven that occurrence of theobromine has limited the direct use of CBS in animal feed, and the European Food Safety Authority (EFSA) has lately established 300 mg/kg as the maximum level of theobromine in feedstuff, with the exception of 700 mg/kg for adult cattle complete feedstuff [105]. For these reasons, several theobromine remediation strategies have appeared in order to increase CBS use as a feeding material. Among these strategies, physicochemical treatments have been proposed, such as the boiling of CBS [143,144] or hydrotropic extraction [144]. Also, several studies have proposed fungi fermentation treatments of CBS for bio-detheobromination, showing that species such as *A. niger*, *Talaromyces*, or *P. ostreatus* spawn are capable of metabolizing theobromine, obtaining up to a 78.13% theobromine content reduction in CBS [69,130,145,146].

### 4.3. Uses in Industry and Other Applications

Besides food applications and uses as a feedstuff supplement, CBS applications are numerous and varied. Among other uses, we can find CBS being used for biofuel production, activated carbon preparation, bioadsorbents, mulch, fertilizer, etc.

CBS have been employed in industry as biomass for fuel production because of its high calorific value that ranges between 7400 and 8600 BTU (British termal unit), being slightly higher than that of wood [47]. Mancini et al. [147] produced biomethane from CBS, obtaining up to 199 mL CH_4_/g volatile solids and increased this value by 14% when pretreating CBS with *N*-methylmorpholine-*N*-oxide. Ilham and Fazil [148] obtained biogas by performing an anaerobic co-digestion of cow manure and CBS, producing 10-fold higher quantities of biogas than with the anaerobic digestion of cow manure alone. Awolu and Oyeyemi [149] used CBS for bioethanol production, using acid hydrolysis and fermentation with *Saccharomyces cerevisae*.

CBS have also been widely used in diverse material production processes, taking advantage of both the chemical and physical properties of CBS. CBS have been incorporated into bioplastics to give them antioxidant properties, biodegradable characteristics, and enhanced physical properties with minimal compound migration (less than the 10 mg/dm^2^ allowed by the European Union for bioplastics) for use in food packaging, cosmetics, or biomedical devices [129,150,151]. Lik et al. [152] developed particleboard, adding up to 60% CBS, while other studies have used the by-product for asbestos substitution in the fabrication of composite brake pads, obtaining good quality materials [153,154]. Also, the addition of CBS to aluminum has been carried out, providing enhanced hardness to the material, although both the tensile strength and ductility were compromised in this case [155].

Due to its particular macromolecular composition, which is rich in lignin, CBS employment for active carbon production has also been extensively proposed. Plaza-Recobert et al. [156] prepared activated carbon monoliths from CBS without a binder, thanks to the by-product’s composition of lignocellulosic molecules, gums, pectin, and fats. Other studies have managed to control the active carbon mesoporosity by employing CBS as a lignocellulosic precursor [157]. Ahmad et al. [158] used CBS-based activated carbon as an efficient cationic dye (methylene blue) absorber. Indeed, several authors have proposed CBS, with or without modifications, as a bioadsorbent for textile dyes, gas pollution, heavy metals, or even protein immobilization [159,160,161].

The utilization of CBS as organic mulch or fertilizer is also very common [162]. Although some authors claim that CBS is too light and that it could affect soil properties if used in large quantities, others consider that its content of nitrogen, phosphate, and potassium could add quality to soil when used as a mulch or fertilizer [123,163]. Furthermore, this cocoa by-product acts as a humus-forming base, since it does not decompose rapidly, making it optimal for use as a fertilizer. Indeed, it has been widely used as a support for fungi cultivation [164,165,166].

Other examples of alternative CBS uses are utilization for endoglucanase production by fermenting it with *Penicillium roqueforti*, which could be of large importance as these cellulase enzymes are widely used in different fields such as the food industry for the extraction of fruit and vegetable juices, in bioethanol production, in paper and cellulose production, or in textile and laundry [167]. Tu et al. [168] developed a natural dye from CBS that was able to give UV protection properties to cotton fabric and Fontes et al. [169] valorized CBS ashes for cement replacement in concrete, obtaining an acceptable and durable material, although its mechanical strength was reduced as a consequence.

## 5. Biofunctionality and Potential Health Benefits

Since first being consumed by humans, several health benefits and different beneficial properties have been attributed to the fruit of the cocoa tree, mainly because of the high content of polyphenols, mostly flavonoids. As previously mentioned, these cocoa phytochemicals have been largely reported to give different biofunctionalities to cocoa products, such as anti-cancer activity [170,171,172], effects against diabetes [86,171], effects against neurodegenerative disorders [84], benefits on cardiovascular health [171], action as antimicrobial agents [173], or properties of inflammatory mediators [174]. Also, cocoa polyphenols are well known for their antioxidant properties, which are in many cases responsible in part for all the previously mentioned functions [175,176], together with their particular structures, which makes them resemble several inhibitors and receptor agonists or antagonists of many cell signaling pathways [84]. As could be expected, these properties could probably be extended to other products coming from the cocoa tree, such as the different cocoa by-products. Concretely, for CBS, the by-product that interests us in this literature review, in the last few years, several studies have proposed alternative uses in both the food and pharmaceutical industries because of the benefits that could be provided. More specifically, antibacterial and antiviral properties, benefits against cardiovascular diseases, anticarcinogenic effects, antidiabetic activities, neuroprotective potential, and anti-inflammatory effects have been reported. These biofunctionalities and their mechanisms of action are summarized in Table 2 and explained in subsequent sections of this article.

### 5.1. Antibacterial Activity and Anticariogenic Effects

The widespread use of antibiotics for both livestock and the human population has led to problems such as bacterial resistance or the reduction of economic profits for farmers. For these reasons, products such as CBS have attracted attention because of their antimicrobial capacities, and therefore, their potential to be used as antibiotic substitutes [189].

For CBS, weak antibacterial activity against *Escherichia coli*, *Staphylococcus aureus*, *Salmonella*, and *Bacillus cereus* has been reported when using acetone, ethanol, methanol, and water extracts, with minimal inhibitory concentrations that ranged between 0.468 and 3.750 mg dry extract/mL [41]. The acetone extract seemed to be the most active, while the water extract was the one with higher minimal inhibitory concentrations. However, the authors stated that no direct correlation was found between the polyphenol concentration and antibacterial activity of the different extracts.

Nevertheless, CBS have been shown to possess stronger antibacterial activities against some type of *Streptococci* bacteria, namely *S. mutans*, a strain involved in the development of dental caries. Ooshima et al. [181] found a considerable reduction of both oral *Streptococci*, *S. mutans*, and *S. sobrinus,* growth rates with ethanolic extracts of CBS and stated a reduction of plaque deposition. The latter effect was due to the inhibition of bacteria glucosyltransferases (GTF) by the CBS extract, and therefore, a reduction of their sucrose-dependent cell adherence [177,190]. The works done by Matsumoto et al. and Osawa et al. [178,179] have also reported the cariostatic effects of CBS due to GTF inhibition, proposing that such inhibition could be caused by high-molecular weight polyphenols, concretely by polymeric epicatechins with C-43 and C-8 intermolecular bonds, estimated to have a molecular weight of 4636 in their acetylated form. Another proposed cause for the inhibition of plaque deposition was the reduction of the hydrophobicity on the cell surface of *S. mutans* caused by polyphenols. Activity against *S. mutans* due to the fatty acids contained in CBS has also been proposed, mainly due to oleic and linoleic acids.

Other authors have taken advantage of the CBS anticariogenic effects to develop different oral hygiene products, such as a toothbrush disinfectant capable of reducing up to 32.25% of the total *S. mutans* [182], or mouthwashes able to reduce the occurrence of these bacteria in saliva to the same levels as 0.2% chlorhexidine, a well-known antiseptic agent [180]. Kwon et al. [191] patented a chewing gum containing a 0.1%–1.0% freeze-dried CBS ethanolic extract, aiming to prevent tooth decay.

### 5.2. Antiviral Properties

The antiviral effects of CBS, mainly against HIV and influenza, have been reported to occur mostly due to the lignin-carbohydrate complexes, rather than other polyphenols with a lower molecular weight, such as tannins or flavonoids [184]. Indeed, Sakagami et al. [183] obtained selectivity indices (SI = 50% cytotoxic concentration, CC_50_/50% effective concentration, EC_50_) of CBS lignin fractions against HIV that ranged from 30 to 10,000, which are surprisingly high values when compared to those of tannins (SI = 1–10), flavonoids (SI = 1), or lignin fractions obtained from other sources, such as cocoa mass (SI = 10–100). These values obtained for CBS were in some cases comparable to those of reverse transcriptase inhibitors. Unten et al. [185] found out that CBS action against HIV was at a maximum when extracts were added to cells at the same time as virus adsorption, and, therefore, the action is not directly related to the virus replication after infection, but that it was instead mostly due to the inhibition of virus absorption. Indeed, they observed that the cytopathic effect on highly HIV susceptible cells was inhibited when treated with 31.2–250 μg dried CBS extract/mL. Additionally, they also observed the inhibition of syncytium formation between infected and uninfected cells, avoiding HIV replication in two different ways. Similarly, it was found that the same CBS lignin fractions were able to inhibit the cytopathic effects produced by the influenza virus [183], an effect that was already reported for condensed tannins [173]. These fractions presented a selectivity index of 155 against the influenza virus and could act synergistically with vitamin C, enhancing its activity.

### 5.3. Action on Cardiovascular Health

Cocoa flavanols have been related to cardiovascular disease prevention thanks to different aspects such as their antioxidant activity on plasma, reducing platelet reactivity, or their anti-inflammatory properties that could decrease the risk of arteriosclerosis or thrombosis [171,192,193]. However, the *in vivo* bioavailability of these compounds when obtained with diet is generally low, and therefore, an important part of the CBS contribution to cardiovascular health takes part in the digestive system. Indeed, some authors have related the beneficial effects of CBS on cardiovascular health with the reduction of atherogenic fat absorption. Thus, Nsor-Atindana et al. [79] found that CBS fiber has a considerably high adsorption capacity for oil and cholesterol, decreasing their bioavailability during the gastrointestinal digestion process. On the other hand, Lecumberri et al. [78] reported *in vivo* hypolipidemic and cholesterol reducing effects of CBS in rats, mainly due to the SDF fraction. Indeed, they found significant reductions of the total and low-density lipoprotein cholesterol when consuming CBS after having followed a cholesterol-rich diet. Moreover, the consumption of CBS fiber also reduced lipidic peroxidation in the serum and liver, probably because of the polyphenolic compounds.

### 5.4. Anticarcinogenic Action

Cocoa polyphenols are known to possess anticarcinogenic properties, mainly because of their potential to reduce excessive oxidative stress, which is characteristic of all the different stages of cancer development and is highly involved in DNA damage that leads to mutation [171]. Flavanols and procyanidins from cocoa have also demonstrated to be implicated in the regulation of different cancer-related signal transduction pathways regarding mutagenesis, tumorigenesis, angiogenesis, or metastasis, among others [172,194,195]. Concerning the anticarcinogenic effects of CBS, some studies have revealed antiproliferative effects of fermented CBS methanolic extracts against breast, liver, colon, lung, and cervical cancer cell lines, although unfermented CBS have not shown such promising results [56,187,188]. However, these studies did not show any positive correlation between the antiproliferative effects and the total phenolic content, which may suggest that antiproliferative activity is subject to just some of the compounds contained within CBS extracts. Indeed, Nsor-Atindana et al. [79] found out that dietary fibers from CBS have the capability of adsorbing and detoxifying bile acids, which are known to cause injury in gastric mucosal epithelial cells, resulting in DNA damage, and therefore, can act as potential carcinogens. Sakagami et al. [184] observed that a 100–1000 μg/mL CBS lignin fraction is able to stimulate the proliferation of human normal gingival fibroblasts (HGF), but not those of the human oral squamous cell carcinoma cell line (HSC-2). On the other hand, Lee et al. [186,196] discovered that polyphenolic extracts and fractions of CBS have inhibitory effects on carcinogenesis. These fractions have been shown to inhibit the proliferation of liver, stomach, and colon cancer cells by suppressing their DNA synthesis, namely, 4-fold higher than vitamin C, which is a well-known chemopreventive dietary compound. Besides, the same fractions had a 10-fold higher ability to reduce the inhibition of the gap junction of intracellular communication (GJIC) than vitamin C, an inhibition that is characteristic of carcinogenesis and usually used as a key biochemical index for this phenomenon. This reduction seemed to increase proportionally with the polyphenolic content.

### 5.5. Antidiabetic Activity

Cocoa flavanols have already been proven to act as chemopreventive agents by helping the prevention or treatment of type 2 diabetes mellitus. They can regulate insulin secretion and protect β-pancreatic cells. They also have an insulin-like activity, and thus, cocoa polyphenols can enhance insulin sensitivity by improving glucose transport to tissues such as skeletal muscle, liver, or adipose tissue, resulting in glycemic control, as well as protecting these tissues from the oxidative and inflammatory damages that are associated to diabetes [86]. Regarding CBS, Rojo-Poveda et al. [13] developed CBS functional beverages with a potential antidiabetic capacity, as they were able to inhibit α-glucosidase, an enzyme involved in glucose degradation. Some of those beverages displayed an effect close to that of acarbose 0.5 mM, which is a drug used to treat diabetes. It has also been found that dietary fiber fractions of the by-product can inhibit α-amylase (another enzyme involved in glucose degradation) and adsorb glucose, retarding its diffusion and dialysis through the intestinal wall, which could lead to antidiabetic properties during the passage of CBS through the human gut when consumed with diet [40,79].

### 5.6. Other Biofunctional Properties

In addition to the already mentioned biofunctions, CBS also present neuroprotective, anti-inflammatory, and anti-obesity properties, among others. Supercritical CO_2_ CBS extracts have been shown to protect human neuroblastoma cells against ischemic damage, although the more active fractions were not the ones presenting the higher antioxidant activity [90]. Also, it was found that CBS contain considerable quantities of phytoprostanes and phytofurans, which are believed to be involved in brain and central nervous system development, as they present cytoprotective activity in immature brain cells. Furthermore, these molecules are likely to play a role in the prevention of metabolic disorders and can exhibit anti-inflammatory, immune function regulation, and apoptosis-inducing activities [76].

Likewise, Rossin et al. [80] found that CBS could play an important role in the treatment of inflammatory bowel diseases (IBD), as phenol-rich CBS fractions are able to inhibit the inflammatory effects caused by oxysterols on intestinal cells (Caco-2). These fractions, with the presence of high amounts of (-)-epicatechin and tannins, fully avoid the production of IL-8, which is a pro-inflammatory cytokine, and prevents exaggerated toll-like receptor (TLR)-mediated immune and inflammatory responses.

An action against obesity and inflammatory-related disorders was also proposed by Rebollo-Hernanz et al. [102]. They found that a freeze-dried aqueous extract of CBS, mainly containing polyphenols, was able to inhibit lipid accumulation and lower pro-inflammatory cytokine production. Moreover, it attenuates inflammation in adipose tissue macrophages and inhibits their activation, and therefore, regulates adipokine secretion, which could otherwise cause mitochondrial dysfunction and insulin sensitivity.

In another study, 10 μg/mL of CBS carbohydrate-rich fractions inhibited the cytotoxicity caused by cigarette smoke on HGF and HSC-2 cells, although higher concentrations seemed to increase it [184].

## 6. Safety Aspects

As a by-product of the cocoa tree, CBS could be subjected to several contamination sources during the farming, production, and manufacturing processes, acquiring different chemical compounds that could be harmful to human health. During farming, CBS, as well as the whole cocoa plant, can be exposed to natural contamination coming from the soil in which it the cocoa tree grows, where the main concern is heavy metal absorption. It can also be exposed to contamination by insecticides used during cultivation, which are largely used to reduce the 30%–40% of global cocoa production that is annually lost to insects and diseases [197]. Unfortunately, insecticides, such as neonicotinoids, mainly concentrate on the outer part of the cocoa bean, i.e., the CBS, but some studies have developed methods for their assessment and clean-up, also recommending a greater efficiency of insecticide application to avoid accumulation to unsafe levels. [12,198]. After harvesting and during the journey to chocolate manufacturing locations, CBS could also be exposed to another kind of contamination, such as polycyclic aromatic hydrocarbons (PAHs), due to inappropriate drying processes near smoke [199], or mold and mycotoxin formation during storage and transport. With this, because of the occurrence and importance of these risk factors, a special focus must be given to heavy metals and mycotoxin accumulation when using CBS.

### 6.1. Heavy Metals

Heavy metals generally consist of non-essential metals with toxic potential for human health. As a plant, the cocoa tree absorbs these metals from contaminated soil and accumulates them, mainly in the fruit, and therefore, also on the outer part of the cocoa bean. Being a protective barrier for the bean, CBS absorb higher quantities of heavy metals, mostly lead and cadmium [113,200,201]. Indeed, some authors have studied the possibilities of using CBS as an adsorbent for heavy metal removal from acidic soils [202,203]. Soil is considered an important metal source, as big parts of cocoa crops are located in volcanic soils, though, atmospheric contamination during cocoa treatment and shipping is believed to be the main source in the case of lead contamination [204]. This contamination is caused by leaded gasoline combustion, which is still commonly used in several cocoa-producing countries, where emissions could be in direct contact with the cocoa shell during the fermentation, sun-drying, and shipping processes of cocoa beans [205]. Assa et al. [206] found lead concentrations in CBS ranging from 5.80 to 11.15 mg/kg, which were 100-fold higher than those observed inside cocoa beans and significantly higher than the 1.00 mg/kg of lead contamination allowed by the European Union in cocoa powder, although these values are highly variable between different studies. Hence, it is of great importance to control the lead content of CBS, especially when using them as a food ingredient, and to concentrate efforts on the mitigation of the primary sources. Concerning cadmium, Lewis et al. [207] found that 18%–56% of its total content on the entire cocoa bean is concentrated in the shell, although they claimed that these values could vary depending on the plant genetics and that a genetic strategy could be used in order to mitigate cadmium concentrations. However, levels of 0.05–0.10 mg per kg of CBS have been found for Cd, which are still far from the 1.5 mg/kg maximum allowed by the Codex Alimentarius for cocoa powder [206,208].

### 6.2. Mycotoxins

Mycotoxins are one of the major safety concerns in cocoa and CBS. These are low-molecular weight toxins that are present in a wide variety of food products, produced by some fungi species from the *Aspergillus* and *Penicillium* genera, which can contaminate cocoa during fermentation, drying, and storage, and are generally thermostable such that they are not completely eliminated during roasting [209,210].

One of the main mycotoxins found in cocoa products is ochratoxin A (OTA), which has been related to nephrotoxic, teratogenic, and immunosuppressive activities, and is classified as a 2B carcinogen (possible human carcinogen) [211]. Studies have demonstrated that the major OTA percentage of the total cocoa bean is contained in CBS, typically about 50%–95%, with concentrations that generally range between 0.13 and 2.01 μg of OTA per kg of CBS, which is still within the acceptable values determined by the European Commission for cocoa beans (less than 2 μg/kg) [36,210,211,212], although other authors have reported values of up to 23.1 μg of OTA per kg of CBS [213]. Aflatoxins have also been detected in cocoa products. Similar to OTA, aflatoxins can produce hepatotoxic, teratogenic, mutagenic, and carcinogenic effects in humans. Concretely, aflatoxins B1, B2, G1, and G2 have been detected in CBS in concentrations that range between 0.01–1.00 μg/kg, <0.03–0.02 μg/kg, <0.03–0.44 μg/kg, and <0.03–0.06 μg/kg, respectively, which are still considered as safe values [36,214]. Another mycotoxin detected in CBS was deoxynivalenol, also known as vomitoxin a it creates nausea, which again was found to be concentrated in CBS more than in cocoa nibs, but still in concentrations without posing a risk to human health [215].

Mycotoxins have been demonstrated to be present in CBS in generally acceptable concentrations. However, these levels can sometimes increase because of different factors. For these reasons, several studies have focused their attention on mycotoxin remediation. Amezqueta et al. [209] eliminated up to 98% of the OTA contained in CBS with a simple chemical method, employing sodium carbonate and potassium carbonate at low concentrations, while Arlorio et al. [30] conducted a Soxhlet extraction with 2-propanol eliminating 70.2% of the OTA contained in the CBS. Aroyeun and Adegoke [216] found an OTA reduction efficiency of 64.3%–95% by employing essential oils, while Manda et al. [217] observed an average OTA decrease of 23.8% with just a controlled roasting process at 140 °C for 30 min. Oduro-Mensah et al. [146] proposed a method employing filamentous fungi that degraded 31%–74% of the OTA contained in cocoa pods. Besides, CBS have been affirmed to be especially susceptible to fungi spoilage, due to the pulp residues that can remain on it after bean separation from the pods and fermentation, thus the occurrence of mycotoxins could also be partially prevented if special attention is directed to bean cleanliness after fermentation [218].

## 7. Conclusions and Future Trends

The bean shell represents one of the main by-products in the cocoa and chocolate industries. Contrary to what happens with the other cocoa by-products, CBS are exported together with the bean, and they are normally discarded at the processing place with all the added costs that this entails, namely, extra weight during transport, disposal cost, and the environmental impact. Although not being used for chocolate production, CBS possess several properties similar to that of cocoa powder, which includes similar volatile and polyphenolic profiles. Therefore, they hold similar organoleptic properties to those of chocolate and several benefits are provided by the polyphenolic compounds found in CBS, mainly catechin, epicatechin, and procyanidins. Compared to the cocoa bean, CBS do not contain large quantities of fat, but they are counterbalanced by a considerably high percentage of dietary fiber. The presence of both methylxanthines, caffeine, and theobromine is also characteristic of CBS, and despite the potential toxicity of theobromine, which could act as an antinutrient in animals, CBS have been largely used for feedstuff, generally after theobromine remediation. CBS have also been employed for diverse industrial applications, such as for biofuel production, as bioadsorbents, fertilizers, mulch, or activated carbon formulations. Nevertheless, one of the most remarkable uses of CBS is as an ingredient in the food industry. In order to valorize this by-product, CBS have been largely proposed for the production of different foodstuffs, generally baked products, with the aim of reducing production costs, giving particular structural properties to the generated products and, mostly, to give an added value to the different foods, thanks to the various beneficial properties for human health that have been attributed to CBS. Indeed, the particular composition of CBS, rich in cocoa-similar polyphenols and dietary fiber, has made them a product of interest for many researchers, who consider CBS to be a potential nutraceutical. Nonetheless, safety aspects, such as mycotoxins or heavy metal occurrence in CBS, remain to be monitored when using them for human consumption, and, in some cases, remediation strategies could be needed.

In the last few years, several studies have proposed new valorization strategies for CBS as a biofunctional by-product with beneficial properties for human health. Studies have revealed that CBS possess antibacterial properties, mostly against *S. mutans*, a bacterial strain involved in dental caries, and also antiviral properties against the human immunodeficiency and the influenza viruses. CBS have also shown hypolipidemic and hypocholesterolemic properties, and thus, the potential to provide beneficial effects to the cardiovascular system. An important focus has been directed to the anticarcinogenic activity of CBS, since they have been shown to present an *in vitro* anti-proliferative action for cancer cells and the inhibition of various characteristic processes of carcinogenesis. The antidiabetic capacity of CBS has also been reported, and it is associated with the capability of the polyphenols of CBS to inhibit different glucose degradation enzymes and the sugar retention of its fiber fractions. Furthermore, other interesting properties for human health have been reported, such as neuroprotective, anti-inflammatory, and anti-obesity potential.

All the mentioned facts about CBS were reviewed in the present work, with a special focus on the nutritive components and biofunctional potential for human health. With this overall view, CBS have been demonstrated to be a promising by-product with several possibilities of valorization. CBS hold great potential as a new functional ingredient or cocoa substitute, thus avoiding the costs of CBS disposal by giving health benefits to the consumer. However, now that these potentials have been investigated and proposed (mostly *in vitro*), more research, and also *in vivo* and clinical studies are necessary to finally confirm and evaluate the biofunctional capacity of this by-product.

## Figures and Tables

**Table 1:** Nutritional and chemical composition of cocoa bean shells (CBS).

Parameter	Amount ^a^	References
Energy (kcal/100 g)	122.00	[15]
Moisture (%)	3.60–13.13	[13,26,27,30,31,32,33,34,35,36,37,38]
Ash (g/100 g)	5.96–11.42	[13,30,33,35,36,37,38,39,40,41]
Proteins (g/100 g)	10.30–27.40	[13,26,27,30,32,34,35,36,37,38,40,41,42,43,44]
Fats (g/100 g)	1.50–8.49	[13,30,32,33,35,37,38,40,41,42,43,44,45]
Carbohydrates (g/100 g)	7.85–70.25	[13,15,32,35,38,42]
- Starch (g/100 g)	0–2.80	[24,27,39,46,47]
- Soluble sugars (g/100 g)	0.16–1.66	[27,32,40]
Dietary fiber (g/100 g)	39.25–66.33	[13,17,30,33,35,38,40,43,48]
- Soluble fiber (g/100 g)	7.03–16.91	[13,17,33,35,36,38,40,41]
- Insoluble fiber (g/100 g)	28.34–50.42	[13,17,33,35,36,38,40,41]
Pectin (g/100 g)	7.62–15.59	[30,49,50,51]
Minerals		
- Calcium (g/100 g)	0.23–0.44	[39,52]
- Phosphorus (g/100 g)	0.58–1.00	[39]
- Magnesium (g/100 g)	0.48–1.29	[39,52]
- Potassium (g/100 g)	1.25–1.82	[39,52]
- Sodium (mg/100 g)	16.00–192.20	[39,52]
- Iron (mg/100 g)	27.60–80.50	[39,52]
- Manganese (mg/100 g)	4.53	[52]
- Copper (mg/100 g)	2.35–6.62	[39,52]
- Selenium (mg/100 g)	0.21	[52]
- Cobalt (mg/100 g)	0.10	[52]
- Zinc (mg/100 g)	2.75–19.00	[39,52,53]
- Chromium (mg/100 g)	0.67‒4.86	[39,52]
Vitamins		
- B1 (μg/g)	0.70–3.10	[39]
- B2 (μg/g)	0.90–3.10	[39]
- B6 (μg/g)	tr	[39]
- D (μg/g)	tr–0.53	[39,54]
- E (μg total tocopherols/g CBS fat)	1.02	[36]
Polyphenol content		
- Total phenolic content ^b^	3.12–94.95	[13,40,41,55,56,57,58,59,60,61]
- Total flavonoid content ^c^	1.65–40.72	[13,41,59]
- Total tannin content ^c^	1.70–25.30	[13,39,41,59]
Flavanols		
- Epicatechin (mg/g)	0.21–34.97	[59,62,63,64,65]
- Catechin (mg/g)	0.18–4.50	[62,63,64,65]
- Procyanidin B1 (mg/g)	0.55–0.83	[65]
- Procyanidin B2 (mg/g)	0.23–1.38	[64,65]
Methylxanthines		
- Theobromine (g/100 g)	0.39–1.83	[30,37,39,47,57,59,62,64,66,67]
- Caffeine (g/100 g)	0.04–0.42	[39,47,57,59,63,64,66]
Volatile organic compounds (aromatics; μg/g)	4.92–16.10	[31,68]

^a^ Data are referred to a CBS dry weight basis unless indicated differently. Intervals have been created, comprising all the values from the cited literature; ^b^ mg of gallic acid equivalents/g of dried CBS; ^c^ mg of catechin equivalents/g of dried CBS; CBS: cocoa bean shell; tr: Traces.

**Table 2:** Preparations, applications, and mechanisms of action of the main CBS biofunctionalities and benefits for human health reported in the literature.

Extract/Fraction (Dose)	Application	Mechanism of Action	References
Acetone, ethanol, methanol (80%), and water extracts of defatted CBS (100 mg dry CBS/mL)	Antibacterial	Weak antibacterial activity against *E. coli, S. aureus, Salmonella,* and *B. cereus* (acetone > ethanol = methanol > water) when compared to a positive control (cephadex) with the inhibition zone diameter technique. Minimum inhibitory concentrations (MIC) of 0.468–3.750 mg/mL when using microdilution technique.	[41]
CBS ethanol, methanol, and acetone (50%, v/v) dried extracts (0.1 g/mL)	Antibacterial (anti-cariogenic)	Inhibitory activity against glucosyltransferase (GTF) from *S. mutans*.	[177]
Cellulase-treated CBS extracted with 30% ethanol, re-dissolved with1% ethanol (1.0 mg dry extract/mL)*—in vitro*	Antibacterial (anti-cariogenic)	*In vitro*: Avoid plaque deposition by inhibiting the adherence of *S. mutans* to saliva-coated hydroxiapatite (inhibition of glucosyltransferase due to epicatechin polymers) and reducing *S. mutans* in plaque (antibacterial activity of unsaturated fatty acids on CBS).	[178]
CBS dissolved in 20% ethanol (2 0mg/mL), diluted with water to 1 mg/mL in 1% ethanol (mouth rinses)—*in vivo*		*In vivo*: CBS mouth rinse more efficient than just 1% ethanol mouth rinse. Inhibition of plaque deposition and *S. mutans* in saliva.	
Cellulase-treated CBS extracted with 50% ethanol and fractionation of the dried extract by chromatography	Antibacterial (anti-cariogenic)	Antibacterial activity due to oleic and linoleic acids on CBS.	[179]
		Inhibition of bacterial adhesion due to the glucosyltransferase inhibition by polymeric epicatechins with C-43 and C-8 intermolecular bonds estimated to be 4636 in molecular weight in an acetylated form.	
0.1% CBS extract mouth rinse prepared as in [178]	Antibacterial (anti-cariogenic)	*In vivo* reduction of *S. mutans* in saliva significantly similar to that obtained with 0.2% chlorexidine.	[180]
Cellulase-treated CBS extracted with 30% ethanol and resuspended on water (1 mg/mL)	Antibacterial (anti-cariogenic)	*In vitro* and *in vivo* reduction of oral *Streptococci* (*S. mutans and S. sobrinus*) growth rate and reduction of plaque deposition by decreasing sucrose-dependent adherence (inhibition of GTF). Minimum cariostatic concentration of 1.0 mg dry extract/mL.	[181]
Cellulase-treated CBS extracted with 50% ethanol and resuspended in water (1 mg/mL)	Antibacterial	CBS extract used as toothbrush disinfectant, reducing up to 32.25% of bacterial contamination by *S. mutans*.	[182]
Lignin fractions of CBS extracted with 1% NaOH and precipitated with acetic acid and ethanol	Antiviral(HIV and influenza)	Carbohydrate-rich fractions showed a high selectivity index against HIV (SI = 30–10000). Inhibition of cytopathic effects produced by the influenza virus against MDCK cells (Madin-Darby Canine Kidney cells).	[183,184]
		Enhanced radical scavenging activity synergistically with vitamin C.	
	Anti-carcinogenic	100–1000 μg/mL of the CBS lignin fraction stimulates the proliferation of human normal gingival fibroblasts (HGFs), but not that of the human oral squamous cell carcinoma cell line (HSC-2).	
CBS extracted with 0.1 N NaOH, then lyophilized and fractionated according to the molecular weight	Antiviral (HIV)	Anti-HIV activity via inhibition of virus adsorption, and, therefore, inhibition of the cytopathic effect on MT-2 and MT-4 cells (highly sensible to HIV-1) when treated with 31.2–250 μg DW CBS/mL. Inhibition of syncytium formation between uninfected and HIV-infected MOLT-4 cells (lymphoblastoid T-cell line).	[185]
Ground CBS (60% of total dietary fiber)	Effects on cardiovascular health	Hypolipidemic and cholesterol reducing action *in vivo*: Reduction of total and low-density lipoprotein cholesterol and reduction of the lipid peroxidation in serum and liver. Mostly soluble fraction of the dietary fiber.	[78]
Fractionated (20%, 40%, 60%, and 80% ethanol) CBS freeze-dried extracts obtained with 50% methanol, ethanol, and acetone (0.1 g/mL)	Anti-carcinogenic	CBS polyphenolic fractions reduce the DNA synthesis of cancer cells and the inhibition of the gap-junction intracellular communication (GJIC).	[186]
Dried methanolic extract of CBS (5 g/200 mL)	Anti-carcinogenic	Anti-proliferative action against breast, liver, colon, lung, and cervical cancer cell lines.	[56,187,188]
Soluble dietary fiber (SDF), insoluble dietary fiber (IDF), and total dietary fiber (TDF) powders from CBS	Anti-carcinogenic	Binding capacity for bile acids (potential carcinogens) resulting in their detoxification.	[79]
	Effects on cardiovascular health	Binding capacity for oil and cholesterol, decreasing their bioavailabilities.	
	Antidiabetic	Absorption of glucose retarding its diffusion and α-amylase inhibition.	
Aqueous extracts of CBS	Antidiabetic	Inhibition of α-glucosidase enzyme close to that of acarbose 0.5 mM.	[13]
Desugared ground CBS	Antidiabetic	Sugar retention on the SDF fraction.	[40]
Supercritical CO_2_ extracts of CBS dissolved in acetone	Neuroprotective action	Protective action against ischemic oxidative damage in neuronal phenotype differentiated cells.	[90]
Dried ground CBS	Neuroprotective action and anti-inflammatory	Great content on phytoprostanes (474.3 ng/g DW CBS) phytofurans (278.0 ng/g DW CBS) with cytoprotective activity in immature brain cells and involved in anti-inflammatory processes.	[76]
Methanol and acetone CBS fractions of ethanolic extract (rich in epicatechin and tannins; 5 mg dried extract/mL)	Anti-inflammatory	Prevention of oxysterol mixture-induced IL-8 release (pro-inflammatory cytokine) on Caco-2 intestinal cell models and prevention of exaggerated toll-like receptor 2 and 4 (TRL2 and TRL4) responses, which may contribute to induce oxysterol-dependent intestinal inflammation.	[80]
Freeze-dried CBS aqueous extract (0.02 g/mL)	Action against obesity and inflammation-related disorders	Adipogenesis modulation and inhibition of adipokine production (responsible for inflammation processes and insulin resistance).	[102]

## Abbreviations

CBS—cocoa bean shell, DW—dry weight, TDF—total dietary fiber, SDF—soluble dietary fiber, IDF—insoluble dietary fiber, TPC—total phenolic content, TFC—total flavonoid Content, TTC—total tannin content, GTF—glucosyltransferase, OTA—ochratoxin A.

## References

[1] González J., Pérez D., Gutiérrez Y.I., Scull R. (2018). Pharmacognostic and Physicochemical Studies of *Theobroma cacao* bean husk in Cuba. Int. Invent.Sci. J..

[2] ICCO (2019). Quarterly Bulletin of Cocoa Statistics (Cocoa year 2018/19).

[3] Alimentarius C. (2016). Standard for cocoa (cacao) mass (cocoa/chocolate liquour) and cocoa cake. Codex Stan.

[4] Quelal-Vásconez M.A., Lerma-García M.J., Pérez-Esteve É., Arnau-Bonachera A., Barat J.M., Talens P. (2019). Fast detection of cocoa shell in cocoa powders by near infrared spectroscopy and multivariate analysis. Food Control.

[5] Beckett S.T. (2018). The Science of Chocolate.

[6] Battegazzore D., Bocchini S., Alongi J., Frache A. (2014). Plasticizers, antioxidants and reinforcement fillers from hazelnut skin and cocoa by-products: Extraction and use in PLA and PP. Polym. Degrad. Stab..

[7] Chandrasekaran M. (2012). Valorization of Food Processing by-Products.

[8] Hashimoto J.C., Lima J.C., Celeghini R.M., Nogueira A.B., Efraim P., Poppi R.J., Pallone J.A. (2018). Quality control of commercial cocoa beans (*Theobroma cacao* L.) by near-infrared spectroscopy. Food Anal. Methods.

[9] Zhao J., Fleet G. (2014). Yeasts are essential for cocoa bean fermentation. Int. J. Food Microbiol..

[10] Gardner R. Nutritional Outlook. https://www.nutritionaloutlook.com/herbs-botanicals/cacao-pulp-its-not-just-waste-product-cocoa-anymore.

[11] Baker T.L. Cocoa Nibs. http://www.thelonebaker.com/journal/2011/9/12/cocoa-nibs.html.

[12] Afrane G., Ntiamoah A. (2011). Use of pesticides in the cocoa industry and their impact on the environment and the food chain. Pesticides in the Modern World-Risks and Benefits.

[13] Rojo-Poveda O., Barbosa-Pereira L., Mateus-Reguengo L., Bertolino M., Stévigny C., Zeppa G. (2019). Effects of particle size and extraction methods on cocoa bean shell functional beverage. Nutrients.

[14] Kofink M., Papagiannopoulos M., Galensa R. (2007). (-)-Catechin in cocoa and chocolate: occurence and analysis of an atypical flavan-3-ol enantiomer. Molecules.

[15] Adamafio N. (2013). Theobromine toxicity and remediation of cocoa by-products: An overview. J. Biol. Sci..

[16] Olaifa F., Hamzat R., Oyetoyan O. (2008). Acute toxicity of ethanol extracts of cocoa bean shell on Sarotherodon galilaeus juveniles. J. Fish. Int..

[17] Martín-Cabrejas M.A., Valiente C., Esteban R.M., Mollá E., Waldron K. (1994). Cocoa hull: a potential source of dietary fibre. J. Sci. Food Agric..

[18] Badrie N., Bekele F., Sikora E., Sikora M. (2015). Cocoa agronomy, quality, nutritional, and health aspects. Crit. Rev. Food Sci. Nutr..

[19] Kowalska H., Czajkowska K., Cichowska J., Lenart A. (2017). What’s new in biopotential of fruit and vegetable by-products applied in the food processing industry. Trends Food Sci. Technol..

[20] Ravindran R., Jaiswal A.K. (2016). Exploitation of food industry waste for high-value products. Trends Biotechnol..

[21] Okiyama D.C.G., Navarro S.L.B., Rodrigues C.E.C. (2017). Cocoa shell and its compounds: Applications in the food industry. Trends Food Sci. Technol..

[22] Panak Balentić J., Ačkar Đ., Jokić S., Jozinović A., Babić J., Miličević B., Šubarić D., Pavlović N. (2018). Cocoa shell: A by-product with great potential for wide application. Molecules.

[23] Thangaraj P. (2016). Proximate Composition Analysis. Pharmacological Assays of Plant-Based Natural Products.

[24] Pérez E., Méndez A., León M., Hernández G., Sívoli L. (2015). Proximal composition and the nutritional and functional properties of cocoa by-products (pods and husks) for their use in the food industry. Cocoa By-Prod. Technol. Rheol. Styl. Nutr..

[25] Diomande D., Antheaume I., Leroux M., Lalande J., Balayssac S., Remaud G.S., Tea I. (2015). Multi-element, multi-compound isotope profiling as a means to distinguish the geographical and varietal origin of fermented cocoa (*Theobroma cacao* L.) beans. Food Chem..

[26] Agus B.A.P., Mohamad N.N., Hussain N. (2018). Composition of unfermented, unroasted, roasted cocoa beans and cocoa shells from Peninsular Malaysia. J. Food Meas. Charact..

[27] Bonvehı J.S., Coll F.V. (1999). Protein quality assessment in cocoa husk. Food Res. Int..

[28] Osundahunsi O., Bolade M., Akinbinu A. (2007). Effect of cocoa shell ash as an alkalizing agent on cocoa products. J. Appl. Sci..

[29] WHO (2011). Quality Control Methods for Herbal Materials.

[30] Arlorio M., Coisson J., Restani P., Martelli A. (2001). Characterization of pectins and some secondary compounds from *Theobroma cacao* hulls. J. Food Sci..

[31] Barbosa-Pereira L., Rojo-Poveda O., Ferrocino I., Giordano M., Zeppa G. (2019). Analytical dataset on volatile compounds of cocoa bean shells from different cultivars and geographical origins. Data Brief.

[32] Vojvodić A., Komes D., Vovk I., Belščak-Cvitanović A., Bušić A. (2016). Compositional evaluation of selected agro-industrial wastes as valuable sources for the recovery of complex carbohydrates. Food Res. Int..

[33] Bonvehí J.S., Benería M.A. (1998). Composition of dietary fibre in cocoa husk. Z. Für Lebensm. Und-Forsch. A.

[34] Mancini G., Papirio S., Lens P.N., Esposito G. (2018). Anaerobic Digestion of Lignocellulosic Materials Using Ethanol-Organosolv Pretreatment. Environ. Eng. Sci..

[35] Martínez R., Torres P., Meneses M., Figueroa J., Pérez-Álvarez J., Viuda-Martos M. (2012). Chemical, technological and *in vitro* antioxidant properties of cocoa (*Theobroma cacao* L.) co-products. Food Res. Int..

[36] Okiyama D.C., Soares I.D., Toda T.A., Oliveira A.L., Rodrigues C.E. (2019). Effect of the temperature on the kinetics of cocoa bean shell fat extraction using pressurized ethanol and evaluation of the lipid fraction and defatted meal. Ind. Crops Prod..

[37] Belitz H.-D., Grosch W., Schieberle P. (2009). Food Chemistry. Cocoa and Chocolate.

[38] Abarca D., Martínez R., Muñoz J.J., Torres M.P., Vargas G. (2010). Residuos de café, cacao y cladodio de tuna: Fuentes promisorias de fibra dietaria. Rev. Tecnológica-Espol.

[39] Bonvehí J.S. (1998). Constituents of cocoa husks. Z. Für Nat. C.

[40] Lecumberri E., Mateos R., Izquierdo-Pulido M., Rupérez P., Goya L., Bravo L. (2007). Dietary fibre composition, antioxidant capacity and physico-chemical properties of a fibre-rich product from cocoa (*Theobroma cacao* L.). Food Chem..

[41] Nsor-Atindana J., Zhong F., Mothibe K.J., Bangoura M.L., Lagnika C. (2012). Quantification of total polyphenolic content and antimicrobial activity of cocoa (*Theobroma cacao* L.) Bean Shells. Pak. J. Nutr..

[42] Belščak-Cvitanović A., Vojvodić A., Bušić A., Keppler J., Steffen-Heins A., Komes D. (2018). Encapsulation templated approach to valorization of cocoa husk, poppy and hemp macrostructural and bioactive constituents. Ind. Crops Prod..

[43] Ogunsipe M., Balogun K., Oladepo A., Ayoola M., Arikewuyo M. (2017). Nutritive value of cocoa bean shell meal and its effect on growth and haematology of weaning rabbits. Niger. J Agric Food Environ..

[44] Sandoval A.J., Barreiro J.A., De Sousa A., Valera D., López J.V., Alejandro J. (2019). Composition and Thermogravimetric Characterization of Components of Venezuelan Fermented and dry Trinitario Cocoa Beans (*Theobroma cacao* L.): Whole Beans, Peeled Beans and Shells. Rev. Técnica De La Fac. De Ing. Univ. Del Zulia.

[45] El-Saied H.M., Morsi M., Amer M. (1981). Composition of cocoa shell fat as related to cocoa butter. Für Ernährungswiss..

[46] Jentzsch P.V., Ciobotă V., Salinas W., Kampe B., Aponte P.M., Rösch P., Popp J., Ramos L.A. (2016). Distinction of Ecuadorian varieties of fermented cocoa beans using Raman spectroscopy. Food Chem..

[47] Nair K.P. (2010). Cocoa (*Theobroma cacao* L.)—The Agronomy and Economy of Important Tree Crops of the Developing World.

[48] Santana D.P., Sanchez J.L.R., Calle J., de Villavicencio M.N., Ortega L.D., Llanes L.H. (2018). Utilización de la cascarilla de cacao como fuente de fibra dietética y antioxidantes en la elaboración de galletas dulces/Use of cocoa bean shell as a source of dietetic fiber and antioxidants in the production of sweet cookies. Cienc. Y Tecnol. De Aliment..

[49] Chan S.-Y., Choo W.-S. (2013). Effect of extraction conditions on the yield and chemical properties of pectin from cocoa husks. Food Chem..

[50] Mollea C., Chiampo F., Conti R. (2008). Extraction and characterization of pectins from cocoa husks: A preliminary study. Food Chem..

[51] Nazaruddin R. (2011). Effect of ammonium oxalate and acetic acid at several extraction time and pH on some physicochemical properties of pectin from cocoa husks (*Theobroma cacao*). Afr. J. Food Sci..

[52] Chung B.Y., Iiyama K., Han K.W. (2003). Food Science; Compositional Characterization Of Cacao (*Theobroma cacao* L.) Hull. J. Appl. Biol. Chem..

[53] Vītola V., Ciproviča I. (2016). The effect of cocoa beans heavy and trace elements on safety and stability of confectionery products. Rural Sustain. Res..

[54] Knapp A.W., Coward K.H. (1935). The vitamin D activity of cacao shell: the effect of the fermenting and drying of cacao on the vitamin D potency of cacao shell. II. The origin of vitamin D in cacao shell. Biochem. J..

[55] Manzano P., Hernández J., Quijano-Avilés M., Barragán A., Chóez-Guaranda I., Viteri R., Valle O. (2017). Polyphenols extracted from *Theobroma cacao* waste and its utility as antioxidant. Emir. J. Food Agric..

[56] Baharum Z., Akim A., Taufiq-Yap Y., Hamid R., Kasran R. (2014). *In vitro* antioxidant and antiproliferative activities of methanolic plant part extracts of *Theobroma cacao*. Molecules.

[57] Grillo G., Boffa L., Binello A., Mantegna S., Cravotto G., Chemat F., Dizhbite T., Lauberte L., Telysheva G. (2019). Cocoa bean shell waste valorisation; extraction from lab to pilot-scale cavitational reactors. Food Res. Int..

[58] Jokić S., Pavlović N., Jozinović A., Ačkar Đ., Babić J., Šubarić D. (2019). High-Voltage Electric Discharge Extraction of Bioactive Compounds from the Cocoa Bean Shell. Chem. Biochem. Eng. Q..

[59] Barbosa-Pereira L., Guglielmetti A., Zeppa G. (2018). Pulsed electric field assisted extraction of bioactive compounds from cocoa bean shell and coffee silverskin. Food Bioprocess Technol..

[60] Mazzutti S., Rodrigues L.G.G., Mezzomo N., Venturi V., Ferreira S.R.S. (2018). Integrated green-based processes using supercritical CO2 and pressurized ethanol applied to recover antioxidant compouds from cocoa (*Theobroma cacao*) bean hulls. J. Supercrit. Fluids.

[61] Pavlović N., Jakovljević M., Miškulin M., Molnar M., Ačkar Đ., Jokić S. (2019). Green extraction techniques of bioactive components from cocoa shell. Croat. J. Food Sci. Technol..

[62] Hernández-Hernández C., Morales-Sillero A., Fernández-Bolaños J., Bermúdez-Oria A., Morales A.A., Rodríguez-Gutiérrez G. (2019). Cocoa bean husk: Industrial source of antioxidant phenolic extract. J. Sci. Food Agric..

[63] Jokić S., Gagić T., Knez Ž., Šubarić D., Škerget M. (2018). Separation of Active Compounds from Food by-Product (Cocoa Shell) Using Subcritical Water Extraction. Molecules.

[64] Okiyama D.C., Soares I.D., Cuevas M.S., Crevelin E.J., Moraes L.A., Melo M.P., Oliveira A.L., Rodrigues C.E. (2018). Pressurized liquid extraction of flavanols and alkaloids from cocoa bean shell using ethanol as solvent. Food Res. Int..

[65] Papillo V.A., Locatelli M., Travaglia F., Bordiga M., Garino C., Coïsson J.D., Arlorio M. (2019). Cocoa hulls polyphenols stabilized by microencapsulation as functional ingredient for bakery applications. Food Res. Int..

[66] Barišić V., Flanjak I., Križić I., Jozinović A., Šubarić D., Babić J., Miličević B., Ačkar Đ. (2019). Impact of high-voltage electric discharge treatment on cocoa shell phenolic components and methylxanthines. J. Food Process Eng..

[67] Hartati I. (2010). Hydrotopic extraction of theobromine from cocoa bean shell. Momentum.

[68] Barbosa-Pereira L., Rojo-Poveda O., Ferrocino I., Giordano M., Zeppa G. (2019). Assessment of volatile fingerprint by HS-SPME/GC-qMS and E-nose for the classification of cocoa bean shells using chemometrics. Food Res. Int..

[69] Bentil J.A. (2012). Enhancement of the nutritive value of cocoa (*Theobroma cacao*) bean shells for use as feed for animals through a two-stage solid state fermentation with *Pleurotus ostreatus* and *Aspergillus niger*. Int. J. Appl. Microbiol. Biotechnol. Res..

[70] Pätzold R., Brückner H. (2006). Gas chromatographic determination and mechanism of formation of D-amino acids occurring in fermented and roasted cocoa beans, cocoa powder, chocolate and cocoa shell. Amino Acids.

[71] González-Alejo F.A., Barajas-Fernández J., Olán-Acosta M.d.l.Á., Lagunes-Gálvez L.M., García-Alamilla P. (2019). Supercritical Fluid Extraction of Fat and Caffeine with Theobromine Retention in the Cocoa Shell. Processes.

[72] Münch M., Schieberle P. (1999). A sensitive and selective method for the quantitative determination of fatty acid tryptamides as shell indicators in cocoa products. Z. Für Lebensm. Und-Forsch. A.

[73] Janßen K., Matissek R. (2002). Fatty acid tryptamides as shell indicators for cocoa products and as quality parameters for cocoa butter. Eur. Food Res. Technol..

[74] Grillo G., Boffa L., Binello A., Mantegna S., Cravotto G., Chemat F., Dizhbite T., Lauberte L., Telysheva G. (2019). Analytical dataset of Ecuadorian cocoa shells and beans. Data Brief.

[75] Lessa O.A., dos Santos Reis N., Leite S.G.F., Gutarra M.L.E., Souza A.O., Gualberto S.A., de Oliveira J.R., Aguiar-Oliveira E., Franco M. (2018). Effect of the solid state fermentation of cocoa shell on the secondary metabolites, antioxidant activity, and fatty acids. Food Sci. Biotechnol..

[76] Ruesgas-Ramón M., Figueroa-Espinoza M.C., Durand E., Suárez-Quiroz M.L., González-Ríos O., Rocher A., Reversat G., Vercauteren J., Oger C., Galano J.-M. (2019). Identification and quantification of phytoprostanes and phytofurans of coffee and cocoa by-and co-products. Food Funct..

[77] Redgwell R., Trovato V., Merinat S., Curti D., Hediger S., Manez A. (2003). Dietary fibre in cocoa shell: characterisation of component polysaccharides. Food Chem..

[78] Lecumberri E., Goya L., Mateos R., Alía M., Ramos S., Izquierdo-Pulido M., Bravo L. (2007). A diet rich in dietary fiber from cocoa improves lipid profile and reduces malondialdehyde in hypercholesterolemic rats. Nutrition.

[79] Nsor-Atindana J., Zhong F., Mothibe K.J. (2012). *In vitro* hypoglycemic and cholesterol lowering effects of dietary fiber prepared from cocoa (*Theobroma cacao* L.) shells. Food Funct..

[80] Rossin D., Barbosa-Pereira L., Iaia N., Testa G., Sottero B., Poli G., Zeppa G., Biasi F. (2019). A Dietary Mixture of Oxysterols Induces *In Vitro* Intestinal Inflammation through TLR2/4 Activation: The Protective Effect of Cocoa Bean Shells. Antioxidants.

[81] Sarah M., Hanum F., Rizky M., Hisham M. Microwave-assisted extraction of pectin from cocoa peel. Proceedings of the IOP Conference Series: Earth and Environmental Science.

[82] Mollea C., Chiampo F. (2019). Valorization of Cocoa Husks: Pectin Recovery. Int. J. Food Sci..

[83] Wollgast J., Anklam E. (2000). Review on polyphenols in *Theobroma cacao*: changes in composition during the manufacture of chocolate and methodology for identification and quantification. Food Res. Int..

[84] Vauzour D., Rodriguez-Mateos A., Corona G., Oruna-Concha M.J., Spencer J.P. (2010). Polyphenols and human health: prevention of disease and mechanisms of action. Nutrients.

[85] Hussain T., Tan B., Yin Y., Blachier F., Tossou M.C., Rahu N. (2016). Oxidative stress and inflammation: what polyphenols can do for us?. Oxidative Med. Cell. Longev..

[86] Martin M.Á., Goya L., Ramos S. (2016). Antidiabetic actions of cocoa flavanols. Mol. Nutr. Food Res..

[87] Rodriguez-Mateos A., Vauzour D., Krueger C.G., Shanmuganayagam D., Reed J., Calani L., Mena P., Del Rio D., Crozier A. (2014). Bioavailability, bioactivity and impact on health of dietary flavonoids and related compounds: an update. Arch. Toxicol..

[88] Vauzour D. (2014). Effect of flavonoids on learning, memory and neurocognitive performance: relevance and potential implications for Alzheimer’s disease pathophysiology. J. Sci. Food Agric..

[89] Neshatdoust S., Saunders C., Castle S.M., Vauzour D., Williams C., Butler L., Lovegrove J.A., Spencer J.P. (2016). High-flavonoid intake induces cognitive improvements linked to changes in serum brain-derived neurotrophic factor: two randomised, controlled trials. Nutr. Healthy Aging.

[90] Arlorio M., Coïsson J., Travaglia F., Varsaldi F., Miglio G., Lombardi G., Martelli A. (2005). Antioxidant and biological activity of phenolic pigments from *Theobroma cacao* hulls extracted with supercritical CO2. Food Res. Int..

[91] Hernández S.M.P., Estévez J.J., Giraldo L.J.L., Méndez C.J.M. (2019). Supercritical extraction of bioactive compounds from cocoa husk: study of the main parameters. Rev. Fac. De Ing. Univ. De Antioq..

[92] Yusof M., Huzaimi A., Abd Gani S.S., Zaidan U.H., Halmi M.I.E., Zainudin B.H. (2019). Optimization of an Ultrasound-Assisted Extraction Condition for Flavonoid Compounds from Cocoa Shells (*Theobroma cacao*) Using Response Surface Methodology. Molecules.

[93] Zhong J.-L., Muhammad N., Gu Y.-C., Yan W.-D. (2019). A simple and efficient method for enrichment of cocoa polyphenols from cocoa bean husks with macroporous resins following a scale-up separation. J. Food Eng..

[94] Bruna C., Eichholz I., Rohn S., Kroh L., Huyskens-Keil S. (2009). Bioactive compounds and antioxidant activity of cocoa hulls (*Theobroma cacao* L.) from different origins. J. Appl. Bot. Food Qual..

[95] Utami R., Armunanto R., Supriyanto S. (2016). Effects of cocoa bean (*Theobroma cacao* L.) fermentation on phenolic content, antioxidant activity and functional group of cocoa bean shell. Pak. J. Nutr.

[96] Hernández-Hernández C., Viera-Alcaide I., Morales-Sillero A.M., Fernández-Bolaños J., Rodríguez-Gutiérrez G. (2018). Bioactive compounds in Mexican genotypes of cocoa cotyledon and husk. Food Chem..

[97] Karim A.A., Azlan A., Ismail A., Hashim P., Abdullah N.A. (2014). Antioxidant properties of cocoa pods and shells. Malays. Cocoa J..

[98] Azizah A., Ruslawati N.N., Tee T.S. (1999). Extraction and characterization of antioxidant from cocoa by-products. Food Chem..

[99] Awarikabey E., Amponsah I.K., Woode M.Y. (2014). The value of the cocoa bean shell (hull) and the effect of various processing methods on the phyto-constituents and antioxidant activity of the nib and shell. J. Nat. Prod. Plant Resour..

[100] Granato D., Shahidi F., Wrolstad R., Kilmartin P., Melton L.D., Hidalgo F.J., Miyashita K., van Camp J., Alasalvar C., Ismail A.B. (2018). Antioxidant activity, total phenolics and flavonoids contents: Should we ban *in vitro* screening methods?. Food Chem..

[101] González J., Coronela L., Lisa L. (2019). UHPLC-DAD-ESI-MS/MS Analysis of Flavonoids from Ethanolic Extracts of *Theobroma cacao* Husk in Cuba. Open Access Libr. J..

[102] Rebollo-Hernanz M., Zhang Q., Aguilera Y., Martín-Cabrejas M.A., de Mejia E.G. (2019). Cocoa Shell Aqueous Phenolic Extract Preserves Mitochondrial Function and Insulin Sensitivity by Attenuating Inflammation between Macrophages and Adipocytes In Vitro. Mol. Nutr. Food Res..

[103] Heck C.I., De Mejia E.G. (2007). Yerba Mate Tea (Ilex paraguariensis): a comprehensive review on chemistry, health implications, and technological considerations. J. Food Sci..

[104] Bispo M.S., Veloso M.C.C., Pinheiro H.L.C., De Oliveira R.F., Reis J.O.N., De Andrade J.B. (2002). Simultaneous determination of caffeine, theobromine, and theophylline by high-performance liquid chromatography. J. Chromatogr. Sci..

[105] Authority E.F.S. (2008). Theobromine as undesirable substances in animal feed-Scientific opinion of the Panel on Contaminants in the Food Chain. EFSA J..

[106] Baggott M.J., Childs E., Hart A.B., de Bruin E., Palmer A.A., Wilkinson J.E., de Wit H. (2013). Psychopharmacology of theobromine in healthy volunteers. Psychopharmacology.

[107] Ashihara H., Sano H., Crozier A. (2008). Caffeine and related purine alkaloids: biosynthesis, catabolism, function and genetic engineering. Phytochemistry.

[108] Zheng X.-Q., Koyama Y., Nagai C., Ashihara H. (2004). Biosynthesis, accumulation and degradation of theobromine in developing *Theobroma cacao* fruits. J. Plant Physiol..

[109] Hue C., Gunata Z., Breysse A., Davrieux F., Boulanger R., Sauvage F.-X. (2016). Impact of fermentation on nitrogenous compounds of cocoa beans (*Theobroma cacao* L.) from various origins. Food Chem..

[110] Júnior P.C.G., dos Santos V.B., Lopes A.S., de Souza J.P.I., Pina J.R.S., Júnior G.C.A.C., Marinho P.S.B. (2020). Determination of theobromine and caffeine in fermented and unfermented Amazonian cocoa (*Theobroma cacao* L.) beans using square wave voltammetry after chromatographic separation. Food Control.

[111] Sansone R., Ottaviani J.I., Rodriguez-Mateos A., Heinen Y., Noske D., Spencer J.P., Crozier A., Merx M.W., Kelm M., Schroeter H. (2017). Methylxanthines enhance the effects of cocoa flavanols on cardiovascular function: randomized, double-masked controlled studies. Am. J. Clin. Nutr..

[112] Coimbra M.C., Jorge N. (2011). Proximate composition of guariroba (Syagrus oleracea), jerivá (Syagrus romanzoffiana) and macaúba (Acrocomia aculeata) palm fruits. Food Res. Int..

[113] Thyssen G.M., Keil C., Wolff M., Sperling M., Kadow D., Haase H., Karst U. (2018). Bioimaging of the elemental distribution in cocoa beans by means of LA-ICP-TQMS. J. Anal. At. Spectrom..

[114] Mandrile L., Barbosa-Pereira L., Sorensen K.M., Giovannozzi A.M., Zeppa G., Engelsen S.B., Rossi A.M. (2019). Authentication of cocoa bean shells by near-and mid-infrared spectroscopy and inductively coupled plasma-optical emission spectroscopy. Food Chem..

[115] Wickramasuriya A.M., Dunwell J.M. (2018). Cacao biotechnology: current status and future prospects. Plant Biotechnol. J..

[116] Kon S.K., Henry K.M. (1935). The effect of feeding cacao shell to cows on the vitamin D content of butter (milk). Biochem. J..

[117] Collar C., Rosell C.M., Muguerza B., Moulay L. (2009). Breadmaking performance and keeping behavior of cocoa-soluble fiber-enriched wheat breads. Food Sci. Technol. Int..

[118] Kārkliņa D., Gedrovica I., Reca M., Kronberga M. (2012). Production of biscuits with higher nutritional value. Proc. Latvian Acad. Sci. Sec. B. Nat. Exact Appl.Sci..

[119] Öztürk E., Ova G. (2018). Evaluation of Cocoa Bean Hulls as a Fat Replacer On Functional Cake Production. Turk. J. Agric. -Food Sci. Technol..

[120] Martínez-Cervera S., Salvador A., Muguerza B., Moulay L., Fiszman S. (2011). Cocoa fibre and its application as a fat replacer in chocolate muffins. LWT-Food Sci. Technol..

[121] Eggen I.B. (1979). Cocoa Shell Extract. US Patent.

[122] Quijano-Aviles M.F., Franco-Agurto G.L., Suárez-Quirumbay K.B., Barragán-Lucas A.D., Manzano-Santana P.I. (2016). Linear programming formulation of a dairy drink made of cocoa, coffee and orange by-products. Emir. J. Food Agric..

[123] Jozinović A., Panak Balentić J., Ačkar Đ., Babić J., Pajin B., Miličević B., Guberac S., Vrdoljak A., Šubarić D. (2019). Cocoa husk application in the enrichment of extruded snack products. J. Food Process. Preserv..

[124] Altin G., Gültekin-Özgüven M., Ozcelik B. (2018). Chitosan coated liposome dispersions loaded with cacao hull waste extract: Effect of spray drying on physico-chemical stability and *in vitro* bioaccessibility. J. Food Eng..

[125] Altin G., Gültekin-Özgüven M., Ozcelik B. (2018). Liposomal dispersion and powder systems for delivery of cocoa hull waste phenolics via Ayran (drinking yoghurt): Comparative studies on in-vitro bioaccessibility and antioxidant capacity. Food Hydrocoll..

[126] Ismail A., Yee C.L. (2006). Antioxidative effects of extracts of cocoa shell, roselle seeds and a combination of both extracts on the susceptibility of cooked beef to lipid oxidation. J. Food Technol..

[127] Hernández-Hernández C., Morales-Sillero A., Fernández-Prior M.Á., Fernández-Bolaños J., de la Paz Aguilera-Herrera M., Rodríguez-Gutiérrez G. (2019). Extra virgin olive oil jam enriched with cocoa bean husk extract rich in theobromine and phenols. LWT.

[128] Handojo L., Cherilisa, Indarto A. (2018). Cocoa bean skin waste as potential raw material for liquid smoke production. Environ. Technol..

[129] Tran T.N., Heredia-Guerrero J.A., Mai B.T., Ceseracciu L., Marini L., Athanassiou A., Bayer I.S. (2017). Bioelastomers based on cocoa shell waste with antioxidant ability. Adv. Sustain. Syst..

[130] Adeyemo G., Ajayi A., Longe O., Olubamiwa O. (2015). Gut morphology and internal organs of broiler birds fed graded levels of bio-detheobrominized cocoa bean shell (CBS) based diets. J. Exp. Agric. Int..

[131] Drolet R., Arendt T., Stowe C. (1984). Cacao bean shell poisoning in a dog. J. Am. Vet. Med Assoc..

[132] Adeyemo G., Ajayi A., Olubamiwa O. (2015). Performance of broilers fed graded levels of bio-detheobrominized cocoa bean shell (CBS) based diets. Am. J. Exp. Agric..

[133] Emiola I., Ojebiyi O., Akande T. (2011). Performance and organ weights of laying hens fed diets containing graded levels of sun-dried cocoa bean shell (CBS). Int.J. Poult. Sci..

[134] Olumide M.D., Akinsoyinu A., Hamzat R.A. (2014). Egg quality characteristics of layers fed raw, fermented and enzyme-treated cocoa bean shell based diets. Pac. J. Sci. Technol..

[135] Oduniyi O.S. Egg weight and shell quality characteristics of laying hens fed with graded levels of cocoa bean shell. Proceedings of the Sixth International Scientific Agricultural Symposium" Agrosym 2015".

[136] Day E.J., Dilworth B.C. (1984). Toxicity of jimson weed seed and cocoa shell meal to broilers. Poult. Sci..

[137] Adeyina A., Apata D., Annongu A., Olatunde O., Alli O., Okupke K. (2010). Performance and physiological response of weaner rabbits fed hot water treated cocoa bean shell-based diet. Res. J. Anim. Vet. Sci..

[138] Ayinde O., Ojo V., Adeyina A., Adesoye O. (2010). Economics of using cocoa bean shell as feed supplement for rabbits. Pak. J. Nutr..

[139] Amin M., Cahyono A. (2016). The Use of Cocoa Bean Waste as a Supplement in Male Bali Cattle Feeding. Proc. Int. sem. LPVT.

[140] Yajima A., Owada H., Kobayashi S., Komatsu N., Takehara K., Ito M., Matsuda K., Sato K., Itabashi H., Sugimura S. (2017). Cacao bean husk: an applicable bedding material in dairy free-stall barns. Asian-Australas. J. Anim. Sci..

[141] Magistrelli D., Zanchi R., Malagutti L., Galassi G., Canzi E., Rosi F. (2016). Effects of cocoa husk feeding on the composition of swine intestinal microbiota. J. Agric. Food Chem..

[142] Bamba Y., Ouattara N., Soro Y., Ouattara A., Yao K., Gourène G. (2014). Evaluation of production efficiency of Nile tilapia (Oreochromis niloticus L.) fed diets containing crop residues in combination with cocoa bean shell and coconut oil cake in Côte d’Ivoire. Livest. Res. Rural Dev..

[143] Olubamiwa O., Ikyo S., Adebowale B., Omojola A., Hamzat R. (2006). Effect of boiling time on the utilization of cocoa bean shell in laying hen feeds. Int. J. Poult. Sci..

[144] Makinde O.J., Okunade S.A., Opoola E., Sikiru A.B., Ajide S.O., Elaigwu S. (2019). Exploration of Cocoa (*Theobroma cacao*) By-Products as Valuable Potential Resources in Livestock Feeds and Feeding Systems. *Theobroma cacao*-Deploying Science for Sustainability of Global Cocoa Economy.

[145] Aromolaran O., Ogunsakin F.M. (2018). Degradation of Theobromine in Cocoa (*Theobroma cacao*) by-products by Fermentation with Aspergillus niger. South Asian J. Res. Microbiol..

[146] Oduro-Mensah D., Ocloo A., Lowor S.T., Mingle C., Okine L.K.-A., Adamafio N.A. (2018). Bio-detheobromination of cocoa pod husks: reduction of ochratoxin A content without change in nutrient profile. Microb. Cell Factories.

[147] Mancini G., Papirio S., Lens P.N., Esposito G. (2016). Effect of N-methylmorpholine-N-oxide pretreatment on biogas production from rice straw, cocoa shell, and hazelnut skin. Environ. Eng. Sci..

[148] Ilham M., Fazil A. (2018). Performance and kinetic study of the anaerobic co-digestion of cocoa husk and digested cow manure with high organic loading rate. INMATEH-Agric. Eng..

[149] Awolu O., Oyeyemi S.O. (2015). Optimization of bioethanol production from cocoa (*Theobroma cacao*) bean shell. Int. J. Curr. Microbiol. App. Sci.

[150] Papadopoulou E.L., Paul U.C., Tran T.N., Suarato G., Ceseracciu L., Marras S., d’Arcy R., Athanassiou A. (2019). Sustainable Active Food Packaging from Poly (lactic acid) and Cocoa Bean Shells. Acs Appl. Mater. Interfaces.

[151] Puglia D., Dominici F., Badalotti M., Santulli C., Kenny J. (2016). Tensile, thermal and morphological characterization of cocoa bean shells (CBS)/polycaprolactone-based composites. J. Renew. Mater..

[152] Lik H. (2006). Development of particleboard from cocoa shells. Malays. Cocoa J..

[153] Olabisi A.I., Adam A.N., Okechukwu O.M. (2016). Development and assessment of composite brake pad using pulverized cocoa beans shells filler. Int. J. Mater. Sci. Appl..

[154] Olabisi A.I. (2016). Development of asbestos-free automotive brake pad using ternary agro-waste fillers. J. Multidiscip. Eng. Sci. Technol. (JMEST).

[155] Olabisi A.I., Boye T.E., Eyere E. (2017). Evaluation of Pure Aluminium Inoculated with Varying Grain Sizes of an Agro-waste based Inoculant. Adv. Sci. Technol. Eng. Syst. J..

[156] Plaza-Recobert M., Trautwein G., Pérez-Cadenas M., Alcañiz-Monge J. (2017). Preparation of binderless activated carbon monoliths from cocoa bean husk. Microporous Mesoporous Mater..

[157] Pérez-Cadenas M., Plaza-Recobert M., Trautwein G., Alcañiz-Monge J. (2018). Development of tailored mesoporosity in carbonised cocoa bean husk. Microporous Mesoporous Mater..

[158] Ahmad F., Daud W.M.A.W., Ahmad M.A., Radzi R. (2012). Cocoa (*Theobroma cacao*) shell-based activated carbon by CO2 activation in removing of Cationic dye from aqueous solution: Kinetics and equilibrium studies. Chem. Eng. Res. Des..

[159] Takam B., Acayanka E., Kamgang G.Y., Pedekwang M.T., Laminsi S. (2017). Enhancement of sorption capacity of cocoa shell biomass modified with non-thermal plasma for removal of both cationic and anionic dyes from aqueous solution. Environ. Sci. Pollut. Res..

[160] Fioresi F., Vieillard J., Bargougui R., Bouazizi N., Fotsing P.N., Woumfo E.D., Brun N., Mofaddel N., Le Derf F. (2017). Chemical modification of the cocoa shell surface using diazonium salts. J. Colloid Interface Sci..

[161] Diaza V.J.M., Nakayoa J.L.J., Benites E. (2018). Use of coffee grind with cocoa shell as the basis for a filter to reduce lead from contaminated water from a river, Peru. Environ. Sci..

[162] International Cocoa Organization Products That can be Made from Cocoa. https://www.icco.org/faq/52-by-products/115-products-that-can-be-made-from-cocoa.html.

[163] Watson R.R., Preedy V.R., Zibadi S. (2013). Chocolate in Health and Nutrition.

[164] Rosmiza M., Davies W., CR R.A., Jabil M., Mazdi M. (2016). Prospects for increasing commercial mushroom production in Malaysia: challenges and opportunities. Mediterr. J. Soc. Sci..

[165] Prathibha V., Sharadraj K., Nidhina K., Hegde V. (2015). Evaluation of locally available substrates for mass production of *Trichoderma*. J. plant. crops.

[166] Silva T., Souza L., Reis N., Assis S., Ferreira M., Oliveira J., Aguiar-Oliveira E., Franco M. (2017). Cultivation of Penicillium roqueforti in cocoa shell to produce and characterize its lipase extract. Rev. Mex. De Ing. Química.

[167] Oliveira P., de Brito A., Pimentel A., Soares G., Pacheco C., Santana N., da Silva E., Fernandes A.d.A., Ferreira M., Oliveira J. (2019). Cocoa shell for the production of endoglucanase by *Penicillium roqueforti A*TCC 10110 in solid state fermentation and biochemical properties. Rev. Mex. De Ing. Química.

[168] Tu C. (2016). Study about Stability of Cacao Husk Pigment and Its Dyeing Properties on Cotton. Key Engineering Materials.

[169] Fontes C., Silva R., Lima P. (2019). Characterization and Effect of Using Bottom and Fly Ashes from Co-combustion of Cocoa Waste as Mineral Addition in Concrete. Waste Biomass Valorization.

[170] Zumbé A. (1998). Polyphenols in cocoa: are there health benefits?. Nutr. Bull..

[171] Martín M.A., Ramos S. (2016). Cocoa polyphenols in oxidative stress: Potential health implications. J. Funct. Foods.

[172] Martin M.A., Goya L., Ramos S. (2013). Potential for preventive effects of cocoa and cocoa polyphenols in cancer. Food Chem. Toxicol..

[173] Daglia M. (2012). Polyphenols as antimicrobial agents. Curr. Opin. Biotechnol..

[174] Sies H., Schewe T., Heiss C., Kelm M. (2005). Cocoa polyphenols and inflammatory mediators. Am. J. Clin. Nutr..

[175] Jalil A.M.M., Ismail A. (2008). Polyphenols in cocoa and cocoa products: is there a link between antioxidant properties and health?. Molecules.

[176] Weisburger J.H. (2001). Chemopreventive effects of cocoa polyphenols on chronic diseases. Exp. Biol. Med..

[177] Kim K.H., Lee K.W., Kim D.Y., Park H.H., Kwon I.B., Lee H.J. (2004). Extraction and fractionation of glucosyltransferase inhibitors from cacao bean husk. Process Biochem..

[178] Matsumoto M., Tsuji M., Okuda J., Sasaki H., Nakano K., Osawa K., Shimura S., Ooshima T. (2004). Inhibitory effects of cacao bean husk extract on plaque formation *in vitro* and *in vivo*. Eur. J. Oral Sci..

[179] Osawa K., Miyazaki K., Shimura S., Okuda J., Matsumoto M., Ooshima T. (2001). Identification of cariostatic substances in the cacao bean husk: their anti-glucosyltransferase and antibacterial activities. J. Dent. Res..

[180] Babu N.V., Vivek D., Ambika G. (2011). Comparative evaluation of chlorhexidine mouthrinse versus cacao bean husk extract mouthrinse as antimicrobial agents in children. Eur. Arch. Paediatr. Dent..

[181] Ooshima T., Osaka Y., Sasaki H., Osawa K., Yasuda H., Matsumura M., Sobue S., Matsumoto M. (2000). Caries inhibitory activity of cacao bean husk extract in in-vitro and animal experiments. Arch. Oral Biol..

[182] Badiyani B.K., Kumar A., Bhat P.K., Sarkar S. (2013). Chocolate disinfectant: effectiveness of cocoa bean husk extract on Streptococcus mutans in used toothbrushes. Int. J. Oral Care Res..

[183] Sakagami H., Satoh K., Fukamachi H., Ikarashi T., Shimizu A., Yano K., Kanamoto T., Terakubo S., Nakashima H., Hasegawa H. (2008). Anti-HIV and vitamin C-synergized radical scavenging activity of cacao husk lignin fractions. In Vivo.

[184] Sakagami H., Matsuta T. (2013). Biological Activity of Cacao Husk and Mass Lignin-Carbohydrate Complexes. Chocolate in Health and Nutrition.

[185] Unten S., Ushijima H., Shimizu H., Tsuchie H., Kitamura T., Moritome N., Sakagami H. (1991). Effect of cacao husk extract on human immunodeficiency virus infection. Lett. Appl. Microbiol..

[186] Lee K.W., Hwang E.-S., Kang N.J., Kim K.H., Lee H.J. (2005). Extraction and chromatographic separation of anticarcinogenic fractions from cacao bean husk. Biofactors.

[187] Zainal B., Abdah M., Taufiq-Yap Y., Roslida A., Rosmin K. (2014). Anticancer agents from non-edible parts of *Theobroma cacao*. Nat. Prod. Chem. Res..

[188] Zainal B., Abdah M., Taufiq Yap Y., Roslida A., Mohd Redzuan S., Kasran R. (2016). Bioactivity-guided fractionation of potent anti-cancer properties from non-edible tissues of **Theobroma cacao**. Malasyan Cocoa J..

[189] Guil-Guerrero J., Ramos L., Moreno C., Zúñiga-Paredes J., Carlosama-Yepez M., Ruales P. (2016). Antimicrobial activity of plant-food by-products: A review focusing on the tropics. Livest. Sci..

[190] Kim D.Y., Park H.J., Park H.H., Kim H.S., Kwon I.B. (2000). Manufacturing Process of Glucosyltransferase Inhibitors from Cacao Bean Husk. U.S. Patent.

[191] Kwon I.B., Park H.H., An B.J. (1990). Chewing Gum Designed to Prevent Tooth Decay by Blending a Soluble Extract of Cacao Bean Husk. U.S. Patent.

[192] Kris-Etherton P.M., Keen C.L. (2002). Evidence that the antioxidant flavonoids in tea and cocoa are beneficial for cardiovascular health. Curr. Opin. Lipidol..

[193] Steinberg F.M., Bearden M.M., Keen C.L. (2003). Cocoa and chocolate flavonoids: implications for cardiovascular health. J. Am. Diet. Assoc..

[194] Yamagishi M., Natsume M., Osakabe N., Nakamura H., Furukawa F., Imazawa T., Nishikawa A., Hirose M. (2002). Effects of cacao liquor proanthocyanidins on PhIP-induced mutagenesis *in vitro*, and *in vivo* mammary and pancreatic tumorigenesis in female Sprague–Dawley rats. Cancer Lett..

[195] Carnésecchi S., Schneider Y., Lazarus S.A., Coehlo D., Gossé F., Raul F. (2002). Flavanols and procyanidins of cocoa and chocolate inhibit growth and polyamine biosynthesis of human colonic cancer cells. Cancer Lett..

[196] Lee H.J., Lee K.W., Kang K.S., Kim D., Park H.H., Lee M.J., Kim H.S., Kwon I.B. (2004). Extracts of Cacao and Cacao Bean Husk with Inhibitory Effects on Carcinogenesis. U.S. Patent.

[197] ICCO, International Cocoa Organization Integrated Management of Cocoa Pests and Pathogens in Africa: Controlling Indigenous Pests and Diseases and Preventing the Introduction of Exogenous Ones. Proceedings of the Report of Project Inception Workshop.

[198] Dankyi E., Carboo D., Gordon C., Fomsgaard I.S. (2015). Application of the QuEChERS procedure and LC–MS/MS for the assessment of neonicotinoid insecticide residues in cocoa beans and shells. J. Food Compos. Anal..

[199] Owusu-Boateng G., Owusu S. (2015). Methods of cocoa harvesting to drying of bean in Ghana and polycyclic aromatic hydrocarbon concentration in the nib and shell of the cocoa bean. Acad. J. Agric. Res..

[200] Bertoldi D., Barbero A., Camin F., Caligiani A., Larcher R. (2016). Multielemental fingerprinting and geographic traceability of *Theobroma cacao* beans and cocoa products. Food Control.

[201] Kruszewski B., Obiedziński M.W., Kowalska J. (2018). Nickel, cadmium and lead levels in raw cocoa and processed chocolate mass materials from three different manufacturers. J. Food Compos. Anal..

[202] Meunier N., Laroulandie J., Blais J., Tyagi R. (2003). Cocoa shells for heavy metal removal from acidic solutions. Bioresour. Technol..

[203] Meunier N., Blais J.-F., Tyagi R.D. (2004). Removal of heavy metals from acid soil leachate using cocoa shells in a batch counter-current sorption process. Hydrometallurgy.

[204] Taylor D.A. (2005). Lead in Cocoa Products: Where Does Contamination Come From.

[205] Rankin C.W., Nriagu J.O., Aggarwal J.K., Arowolo T.A., Adebayo K., Flegal A.R. (2005). Lead contamination in cocoa and cocoa products: isotopic evidence of global contamination. Environ. Health Perspect..

[206] Assa A., Noor A., Yunus M., Djide M. (2018). Heavy metal concentrations in cocoa beans (**Theobroma cacao** L.) originating from East Luwu, South Sulawesi, Indonesia. Proceedings of Journal of Physics: Conference Series.

[207] Lewis C., Lennon A.M., Eudoxie G., Umaharan P. (2018). Genetic variation in bioaccumulation and partitioning of cadmium in *Theobroma cacao* L. Sci. Total Environ..

[208] FAO/WHO (2018). Codex commitee on contaminants in foods. Proposed draft maximum levels for cadmium in chocolate and cocoa-derived products (at step 4).

[209] Amézqueta S., Gonzalez-Penas E., Lizarraga T., Murillo-Arbizu M., De Cerain A.L. (2008). A simple chemical method reduces ochratoxin A in contaminated cocoa shells. J. Food Prot..

[210] Copetti M.V., Iamanaka B.T., Nester M.A., Efraim P., Taniwaki M.H. (2013). Occurrence of ochratoxin A in cocoa by-products and determination of its reduction during chocolate manufacture. Food Chem..

[211] Coulibaly A., Biego G.H.M., Dembele A., Bohoussou K.M., Toure A. (2013). Cocoa beans and cocoa derivatives from Cote-D’Ivoire: investigating ochratoxin a level and assessing dietary intake adults. Sustain. Agric. Res..

[212] Amezqueta S., Gonzalez-Penas E., Murillo M., Lopez de Cerain A. (2005). Occurrence of ochratoxin A in cocoa beans: effect of shelling. Food Addit. Contam..

[213] Serra Bonvehí J. (2004). Occurrence of ochratoxin A in cocoa products and chocolate. J. Agric. Food Chem..

[214] Copetti M.V., Iamanaka B.T., Pereira J.L., Lemes D.P., Nakano F., Taniwaki M.H. (2012). Determination of aflatoxins in by-products of industrial processing of cocoa beans. Food Addit. Contam. Part A.

[215] Raters M., Matissek R. (2008). Analysis and occurrence of deoxynivalenol (DON) in cocoa. Eur. Food Res. Technol..

[216] Aroyeun S., Adegoke G. (2007). Reduction of ochratoxin A (OTA) in spiked cocoa powder and beverage using aqueous extracts and essential oils of Aframomum danielli. Afr. J. Biotechnol..

[217] Manda P., Dano D.S., Kouadio J.H., Diakite A., Sangare-Tigori B., Ezoulin M.J.M., Soumahoro A., Dembele A., Fourny G. (2009). Impact of industrial treatments on ochratoxin A content in artificially contaminated cocoa beans. Food Addit. Contam..

[218] Kreibich H., Moecke E.O.E., Scussel V., Méndez-Vilas A. (2017). Stereo and scanning electron microscopy of cocoa beans (**Theobroma cacao** L.): fungi spoilage susceptibility. Microscopy and Imaging Science: Practical Approaches to Applied Research and Education.

